# CNT Localization and Network Formation in High-Performance PEEK/PEI Blends and Its Effect on Electrical Conductivity

**DOI:** 10.3390/polym18161972

**Published:** 2026-08-13

**Authors:** Behnam Khaledi, Nicole R. Demarquette, Eric David

**Affiliations:** Department of Mechanical Engineering, École de Technologie Supérieure, Montreal, QC H3C 1K3, Canada; nicoler.demarquette@etsmtl.ca (N.R.D.); eric.david@etsmtl.ca (E.D.)

**Keywords:** polymer blend, polymer nanocomposite, PEEK, conductive nanocomposite, double percolation, space-grade polymer

## Abstract

High-performance thermoplastics are gaining increasing attention for space applications. However, the extreme lunar environment requires multifunctional materials that combine electrical conductivity for electrostatic charge dissipation with low thermal conductivity for thermal insulation. One promising strategy to achieve this balance is through conductive polymer nanocomposites with controlled morphology. In this study, the localization, migration, and network formation of carbon nanotubes (CNTs) in two-phase blends of polyetheretherketone/polyetherimide (PEEK/PEI) were systematically investigated to establish the relationships between processing, morphology, and the resulting electrical and thermal properties. Despite the strong thermodynamic preference of CNTs for the PEI phase, both PEEK/CNT and PEEK/PEI/CNT nanocomposites exhibit similar electrical percolation thresholds (0.25–0.5 wt.%), attributed to spatial confinement arising from PEEK crystallinity in PEEK/CNT and from phase-selective localization in the blend system, which limits the effective volume available for CNT dispersion. Morphological characterization confirmed co-continuous blend structures and complete CNT migration into the PEI phase in the PEEK/PEI/CNT system, while rheological studies revealed percolated networks forming below the electrical percolation threshold. Processing conditions strongly impacted conductivity: short mixing times preserved interconnected CNT agglomerates and enhanced conductivity, whereas prolonged mixing promoted dispersion but destroyed conductive pathways. Furthermore, thermal annealing induced agglomeration and weakened networks in PEEK/CNT systems but had a negligible effect on PEEK/PEI/CNT composites due to improved CNT–PEI compatibility. Finally, thermal conductivity remained low across all systems, maintaining the material’s insulating performance for the harsh thermal environment of the Moon.

## 1. Introduction

The space industry has traditionally relied on metallic materials, such as aluminum, due to their predictable behavior across wide temperature ranges and their resistance to outgassing. These characteristics have made metals a reliable choice for many space applications. However, emerging mission profiles, particularly those involving lunar environments, introduce new thermal challenges that require reconsideration of material selection. On the Moon, a single night lasts approximately two terrestrial weeks, during which temperatures can drop to around −200 °C. Under such extreme conditions, minimizing heat loss becomes critical for maintaining the internal thermal stability of spacecraft systems [1,2,3]. In this context, materials with low thermal conductivity become highly desirable, and polymers have a clear advantage. Among them, high-performance thermoplastics are gaining increasing attention, as they offer a lightweight alternative with excellent performance under harsh mechanical and thermal conditions [4]. Polyetheretherketone (PEEK) is a high-performance thermoplastic distinguished by its superior mechanical and thermal properties, high chemical resistance, and excellent wear behavior owing to its semi-crystalline structure and rigid molecular backbone composed of alternating ketone and ether linkage. Despite its many advantages, PEEK is inherently electrically insulating, similar to most polymers, with an electrical conductivity as low as 10^−17^ S/cm [5], which restricts its applicability in certain space technologies. To enhance its electrical performance, researchers have studied the incorporation of conductive nanomaterials such as carbon nanotubes (CNTs), which possess exceptional electrical properties, into PEEK matrices using different techniques, including melt mixing [4,6]. However, nanofillers like CNT often also tend to increase thermal conductivity, an effect undesirable in some applications. For example, when designing thermoplastic panels for a lunar rover, the material must provide sufficient electrical conductivity for electrostatic charge dissipation [7,8], while maintaining thermal insulation to protect internal components like battery and electronics from extreme lunar temperatures [3,9].

One strategy to achieve high electrical conductivity without compromising thermal insulation is to design conductive nanocomposites with a low electrical percolation threshold, enabling the formation of a conductive network at minimal filler content, thereby reducing phonon-mediated heat conduction [10]. In addition to achieving a favorable ratio of electrical to thermal conductivity, lowering the percolation threshold also improves processability, reduces fabrication costs, and allows better control over mechanical properties [11,12,13,14]. In the limited number of studies on the electrical properties of PEEK/CNT nanocomposites, a relatively wide range of percolation threshold values have been reported, typically ranging between 1 and 4 wt.% [4,15,16,17,18]. These percolation threshold values seem relatively high compared to those of commodity polymer systems.

A well-established approach to reduce the percolation threshold in polymer nanocomposites is the “double percolation” phenomenon, which involves using a multiphase polymer blend with co-continuous morphology to promote selective filler localization resulting in the formation of the conductive network at lower filler loadings [11,12,13,14]. Blending PEEK with another high-performance polymer, such as polyetherimide (PEI), offers a promising route to achieve such a structure. This approach can yield a matrix with a higher glass transition temperature (Tg) and improved processability, while maintaining strong mechanical performance at a reduced overall material cost compared to neat PEEK [19]. The diamine groups in PEI can adopt meta (m-PEI) or para (p-PEI) conformations, which influence the polymer’s blending behavior. Studies show that the free energy of mixing between PEEK and p-PEI is higher than with m-PEI, leading to immiscibility (or partial miscibility) in the PEEK/p-PEI blend and miscibility in the PEEK/m-PEI one [20,21,22,23]. Accordingly, using p-PEI is preferable when the goal is to obtain a co-continuous morphology.

Research on PEEK/PEI blends remains relatively limited, with only a small number of studies specifically focusing on PEEK/p-PEI [19,20]. To date, no investigation has reported electrically conductive nanocomposites based on PEEK/p-PEI systems, revealing a significant gap in the current literature. A closely related system was investigated by Gao and co-workers, who employed thermoplastic polyimide (TPI), a polymer with a chemical structure similar to PEI, as the second phase in immiscible PEEK/TPI blends filled with MWCNTs. Their studies demonstrated that the selective localization of CNTs within the TPI phase promoted the formation of a co-continuous morphology and enabled a double-percolation conductive network, reducing the electrical percolation threshold to approximately 0.6 wt.% compared with about 1.3 wt.% for binary PEEK/MWCNT composites. Consequently, the ternary PEEK/TPI/MWCNT nanocomposites exhibited electrical conductivities approximately two orders of magnitude higher than the corresponding PEEK/MWCNT composites. They further showed that the electrical conductivity strongly depended on blend morphology, reaching a maximum at a 50/50 PEEK/TPI composition, where a co-continuous structure was obtained, and also demonstrated that the same morphology significantly enhanced the dielectric properties of the composites [24,25,26].

Although these studies established the effectiveness of the double-percolation concept in high-performance PEEK-based blends, they primarily focused on demonstrating improvements in electrical and dielectric properties resulting from selective CNT localization and optimized blend morphology. The mechanisms governing conductive network formation were not investigated in detail. In particular, the kinetics of CNT migration between polymer phases during melt processing, the evolution of conductive pathways with mixing time, the influence of the filler incorporation sequence, and the effect of post-processing thermal annealing on network stability were not addressed. These processing-dependent phenomena are critical for understanding and controlling the structure–property relationships in conductive PEEK-based nanocomposites.

The objective of the present study is therefore to investigate electrically conductive PEEK/p-PEI nanocomposites and to elucidate the mechanisms governing CNT localization and conductive network formation. In addition to evaluating the effectiveness of the double-percolation approach in reducing the electrical percolation threshold, this work systematically examines the evolution of CNT dispersion and localization in both PEEK/CNT and PEEK/p-PEI/CNT systems. Particular emphasis is placed on the effects of mixing time, CNT migration kinetics, filler incorporation sequence, and thermal annealing on conductive network development and the resulting electrical and thermal transport properties.

## 2. Materials and Methods

### 2.1. Materials

Pellets of PEEK/CNT masterbatch (Plasticyl PEEK 1001), containing 10 wt.% of MWCNT NC7000, were purchased from Nanocyl (Sambreville, Belgium). The masterbatch was diluted to produce nanocomposites with lower CNT content. Polyetherimide pellets (Ultem CRS 5001, referred to as para-PEI or p-PEI) were obtained from SABIC (Riyadh, Saudi Arabia). The chemical structure of Ultem CRS 5001, based on a para-diamine configuration, was initially characterized by other authors [20,27]. A summary of their key properties is presented in Table 1 and Table 2.

### 2.2. Blend Preparation

Nanocomposites of PEEK/CNT, PEI/CNT, and PEEK/p-PEI/CNT were prepared via melt mixing. All polymers were first dried overnight at 150 °C to remove residual moisture. The dried materials were then processed using a Haake Minilab 2 micro-compounder (Thermo Fisher Scientific, Waltham, MA, USA), operating at a temperature of 375 °C and a screw speed of 100 rpm. After all components were introduced, mixing was performed for 10 min in continuous cycling mode to ensure uniform dispersion. Upon completion, the resulting material was extruded as a filament and cooled to room temperature. For selected samples, the mixing time was varied to investigate its influence on the final properties; specific mixing times are provided in the relevant sections. The PEEK:PEI ratio in blends is 40:60 wt.%, which, as shown in the previous study on the same blend, gives co-continuous morphology [19], and the CNT contents in all samples are 0.25, 0.5, 0.75, and 1 wt.%.

### 2.3. Thermal Analysis

Differential scanning calorimetry (DSC, TA Instruments 2500, Waters, Milford, MA, USA) was used to characterize both the neat polymers and the fabricated nanocomposites. The thermal analysis followed a two-cycle heat/isotherm/cool procedure: (1) the samples were first heated from 20 °C to 400 °C at a rate of 10 °C/min, held isothermally for 1 min at 400 °C, and then cooled back to 20 °C at the same rate; (2) a second heating cycle to 400 °C was performed under identical conditions, followed by another isothermal hold at 400 °C for 1 min and subsequent cooling to 20 °C. T_g_ was determined from the inflection points of the baseline during the second heating cycle according to ASTM E1356. The degree of crystallinity (χ_c_), relative to the total mass of the sample, was calculated using Equation (1).

(1) $$\chi_{c}=\frac{{\Delta H}_{m}-{\Delta H}_{cc}}{{\Delta H}_{f}}$$

Here, ΔH_m_ refers to the specific enthalpy of melting from the second heating cycle to eliminate the effects of any thermal history or prior transitions that may have occurred during sample processing, ΔH_cc_ corresponds to the specific enthalpy of cold crystallization observed during heating, and ΔH_f_ represents the theoretical enthalpy of fusion for 100% crystalline PEEK, taken as 130 J/g [28]. Additionally, the degree of crystallinity attributed solely to the PEEK phase in blend samples (χ_PEEK_) was calculated using Equation (2):

(2) $$\chi_{c}=\frac{{\Delta H}_{m}-{\Delta H}_{cc}}{{\Delta H}_{f}⨯\omega_{PEEK}}$$

Here, ω_PEEK_ represents PEEK’s mass fraction within the blend.

### 2.4. Gravimetric Analysis

To assess the continuity of PEI in the blend samples, a gravimetric method was employed involving selective solvent extraction. Five small specimens were first dried at 150 °C for 24 h to remove any moisture and then weighed. The dried samples were immersed in a solvent mixture comprising 80 vol.% 1-methyl-2-pyrrolidone (NMP), 10 vol.% ethanolamine, and 10 vol.% distilled water, and were gently stirred at 120 °C for few hours, allowing the PEI to be selectively dissolved [19]. Following this extraction process, the specimens were rinsed sequentially with acetone and 95% ethanol to remove residual solvent. They were then subjected to a second drying step at 150 °C for another 24 h before being reweighed. Finally, the continuity of the PEI phase was determined using Equation (3).

(3) $$\mathrm{PEI}\;\mathrm{continuity}\;(\%)=\frac{m_{i}-m_{f}}{m_{i}⨯0.6}⨯100$$

Here, m_i_ is the initial mass of the sample prior to extraction, m_f_ is the mass following extraction, and the constant 0.6 represents the mass fraction of PEI in the blend before extraction.

### 2.5. Morphological Analysis

#### 2.5.1. SEM

The microstructure of the blend samples was examined using scanning electron microscopy (SEM, SU8230 Hitachi, Hitachi High-Tech, Tokyo, Japan). Samples were cryo-fractured in liquid nitrogen to preserve their morphology. To enhance imaging contrast, the PEI phase was selectively removed according to the procedure outlined in Section 2.4. Prior to imaging, an approximately 4 nm platinum coating was applied using a platinum sputter coater system (Q150T Quorum, Quorum Technologies, Laughton, UK). Observations were conducted at an accelerating voltage of 5 kV and a beam current of 11,000 nA. Phase size measurements were performed using ImageJ 1.54 by directly measuring and averaging the diameters of the phases across 30 different regions from 3 different images.

#### 2.5.2. TEM

The nanoscale morphology, spatial dispersion, and localization of CNTs within the nanocomposite samples were investigated using transmission electron microscopy (TEM, JEOL JEM-F200, JEOL, Tokyo, Japan). Prior to TEM analysis, ultrathin sections of the samples, on the order of a few nanometers in thickness, were prepared via ultramicrotomy using a diamond knife at room temperature. This technique enabled the production of smooth, electron-transparent slices suitable for high-resolution imaging. Imaging was performed at an accelerating voltage of 200 kV.

### 2.6. Rheological Measurements

Disks of the samples (25 mm in diameter, 1 mm thick) were prepared by compression molding the extruded materials at 375 °C under a pressure of 5 MPa for 10 min using a high-temperature hydraulic press. Small-amplitude oscillatory shear (SAOS) tests were conducted at 375 °C under nitrogen atmosphere using an MCR 501 rheometer (Anton Paar, Graz, Austria) with a parallel plate geometry (25 mm diameter). A shear strain of 0.3% was used for all samples, as determined to be within the linear viscoelastic region from the dynamic strain sweep test. Time sweep tests were performed on neat PEEK and PEI for 1 h to confirm that sample degradation during the measurements was negligible.

### 2.7. Electrical Conductivity

Bulk electrical conductivity measurements were conducted on disk-shaped samples with a diameter of 25 mm and a thickness of 1 mm. The measurements were performed in alternating current (AC) mode using a Broadband Dielectric Spectrometer (BDS, Novocontrol Technologies, Montabaur, Germany), which allows for precise evaluation of electrical properties over a broad frequency range. To reduce contact resistance and ensure reliable electrical contact, both faces of each sample were coated with a 15 mm diameter thin gold layer via sputtering. The gold-coated samples were then placed between two 20 mm diameter gold-plated electrodes configured for high-accuracy impedance analysis. The AC conductivity was measured over a frequency range from 10^6^ Hz to 0.01 Hz. The electrical conductivity values reported in this study correspond to the real part of complex conductivity at the lowest measured frequency (0.01 Hz). As a result, these values tend to overestimate the actual conductivity, especially when below the percolation threshold. However, once the percolation threshold is surpassed, they provide a close approximation of the true DC conductivity.

### 2.8. Thermal Conductivity

Thermal conductivity measurements were carried out on disk-shaped samples with a thickness of 2 mm using a C-Therm thermal conductivity analyzer (C-Therm Technologies, Fredericton, NB, Canada) based on the Modified Transient Plane Source (MTPS) technique. To improve thermal contact between the sensor and the sample’s surface, a single droplet of distilled water was applied as a contact agent. Each sample was evaluated five times to ensure reproducibility and reduce statistical error, and the average value of the five measurements is reported as the representative thermal conductivity.

## 3. Results and Discussion

### 3.1. Material Characterization

The morphology and, consequently, the electrical and thermal properties of nanocomposites and polymer blends are largely governed by thermodynamic factors. Among these, the surface tension of the individual components influences interfacial tension, which in turn governs the interactions and compatibility between phases [29,30,31,32,33,34]. A lower interfacial tension between the filler and a specific polymer phase typically enhances dispersion and promotes preferential localization of the filler within that phase in multiphase polymer blends [31,35,36]. Therefore, understanding the surface energies of PEEK, PEI, and CNTs is essential for predicting phase-selective filler distribution, which directly impacts the formation of conductive pathways and the overall performance of the nanocomposite.

The thermodynamic preference of nanoparticles in a polymer nanocomposite with a two-phase polymeric matrix can be evaluated by calculating the wetting parameter (ωₐ), derived from Young’s equation: [31]

$$\omega_{a}=\frac{\gamma_{filler-polymerA}-\gamma_{filler-polymerB}}{\gamma_{AB}}$$

The symbols *γ*_*filler*−*polymerB*_ and *γ*_*filler*−*polymerA*_ represent the interfacial energies between the filler and Polymer B, and between the filler and Polymer A, respectively. The symbol *γ*_AB_ represents the interfacial energy between Polymer A and Polymer B. The wetting coefficient reflects the thermodynamic tendency of the filler to localize in a specific area of an immiscible blend, which corresponds to the minimization of relevant interfacial tensions. The predicted localization of the nanoparticles based on the wetting parameter is summarized in Table 3 [31].

Table 4 presents the surface tension and interfacial tension values of the components used in this study at room temperature. Some values were obtained from the literature, while others were calculated using the harmonic mean equation [32,37]:

$$\gamma_{AB}=\gamma_{A}+\gamma_{B}-4(\frac{\gamma_{A}^{d}\gamma_{B}^{d}}{\gamma_{A}^{d}+\gamma_{B}^{d}}+\frac{\gamma_{A}^{p}\gamma_{B}^{p}}{\gamma_{A}^{p}+\gamma_{B}^{p}})$$

The variables *γ*_A_ and *γ*_B_ represent the surface tension of Polymers A and B, respectively. Meanwhile, *γ*_A_^*d*^, *γ*_B_^*d*^, *γ*_A_^*p*^, and *γ*_B_^*p*^ refer to the dispersive and polar components of the surface tensions of Polymers A and B, respectively.

The interfacial tension between PEI and CNTs is lower than that between PEEK and CNTs because of the stronger molecular interactions between PEI and the nanotubes. The aromatic imide groups in PEI can form favorable π–π interactions with the graphitic surface of CNTs, reducing the PEI–CNT interfacial tension and increasing their thermodynamic compatibility [40,41,42]. Although PEEK also contains aromatic rings, its ether–ketone backbone lacks the electron-deficient imide groups that promote these interactions, resulting in a weaker affinity for CNTs [43]. Nevertheless, because the dispersive component dominates the interfacial tension in both polymer/CNT pairs, these differences in molecular interactions produce only a modest difference between the CNT–PEEK and CNT–PEI interfacial tensions.

Despite this modest difference, CNT localization is strongly influenced because the interfacial tension between PEEK and p-PEI is very low (0.14 mN/m). According to the wetting coefficient equation (Equation (1)), the small PEEK–PEI interfacial tension in the denominator amplifies the effect of the modest difference between the CNT–PEEK and CNT–PEI interfacial tensions, producing a large absolute value of the wetting coefficient and a strong thermodynamic driving force for CNT localization in the PEI phase.

Using the interfacial tension values listed in Table 4, a wetting coefficient of −5.3 was calculated for the PEEK/PEI/CNT nanocomposite. Since the wetting coefficient is lower than −1, the calculation predicts complete thermodynamic localization of CNTs in the PEI phase, consistent with the thermodynamic preference of CNTs for PEI [19].

### 3.2. Thermal Analysis

Table 5 presents the glass transition temperature (T_g_), heat of fusion, and degree of crystallinity (χ_c_) of compression-molded samples obtained from the second heating cycle of DSC analysis to eliminate the effects of any thermal history or prior transitions that may have occurred during sample processing. It is noteworthy that PEEK is a semi-crystalline polymer characterized by a distinct melting point, whereas PEI is fully amorphous and lacks a defined melting temperature.

In both heating cycles of the DSC curves for the blend samples, two distinct glass transitions were observed, corresponding to the two polymer phases. As shown in Table 5, the T_g_ of PEEK and PEI are 146 °C and 228 °C, respectively, whereas in the PEEK/p-PEI blend, these values are 166 °C and 205 °C. This indicates a significant upward shift in the T_g_ of PEEK and a downward shift for PEI. Such changes reflect the partial miscibility between PEEK and PEI phases. The phases are not fully immiscible; rather, limited interdiffusion occurs at the interface, such that the PEEK phase contains a small amount of PEI and vice versa [19]. The addition of CNT appears to have no significant effect on the T_g_ of the matrix in either PEEK/CNT or PEEK/PEI/CNT samples.

The most critical finding obtained from the DSC analysis is the degree of crystallinity, as it indicates the fraction of the polymer matrix inaccessible to CNTs. For compression-molded neat PEEK, the crystallinity is 36%. Incorporation of CNTs results in negligible changes to this value, consistent with previous reports on PEEK/CNT nanocomposites [44,45]. This behavior likely arises from the competition between two opposing effects: (1) CNTs act as heterogeneous nucleating agents that promote crystallization, and (2) they restrict polymer chain mobility due to spatial confinement. At the CNT concentrations used, the chain restriction effect appears to balance the nucleation enhancement [44,45,46,47]. Another contributing factor is the tendency of CNTs to form agglomerates in the PEEK, which reduces their effectiveness as nucleating agents compared to when they are well-dispersed as individual nanoparticles [44,45].

Another notable observation from the DSC analysis is the effect of the thermal annealing. Annealing at temperatures above the melting point has a negligible effect on the crystallinity of the samples. This is expected, as heating above the melting point eliminates all crystalline regions. Upon subsequent cooling, recrystallization occurs, but the degree of crystallinity depends mainly on the cooling rate and conditions, which were identical for both annealed and non-annealed samples, resulting in similar crystallinity values. This outcome is advantageous, as the objective of annealing in this study was to examine its influence on CNT network morphology and potential phase coarsening rather than on the crystallinity of the PEEK phase.

**Figure 1:**
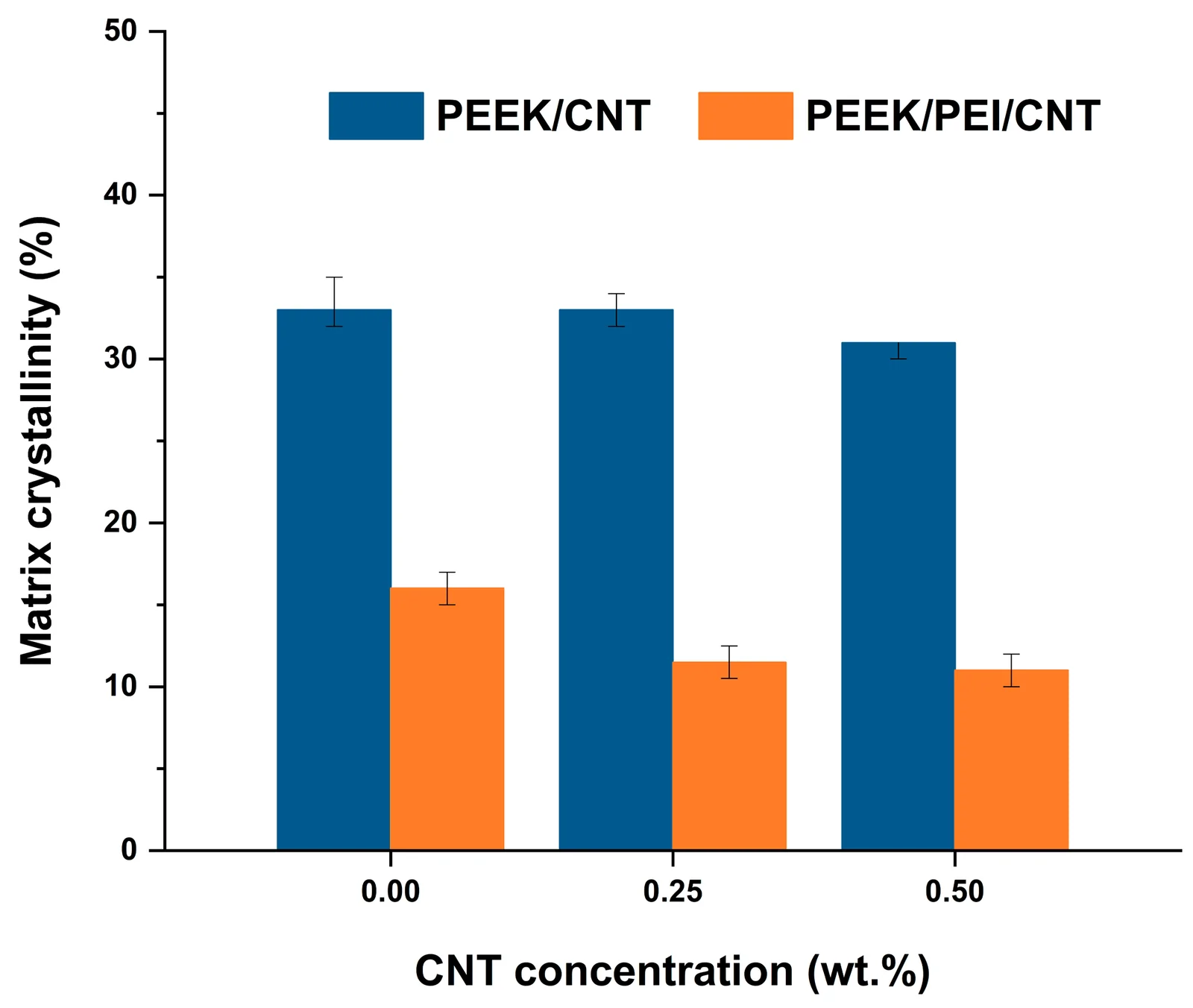
The crystallinity of the matrix in PEEK/CNT and PEEK/PEI/CNT nanocomposites.

Figure 1 presents the crystallinity values of the matrix in PEEK/CNT and PEEK/PEI/CNT samples. Due to both the lower PEEK content in the blend (40 wt.%) and the reduced crystallinity of the PEEK phase after blending (as shown in Table 5), the overall matrix crystallinity in the PEEK/PEI/CNT system decreases by 67% compared to PEEK/CNT. This reduction is potentially advantageous for additive manufacturing applications, including the target application of this study—the fabrication of a lunar rover component—since excessive crystallinity often leads to warpage, anisotropic shrinkage, and, in some cases, poor interlayer adhesion [48,49,50].

### 3.3. Blend Characterization

**Figure 2:**
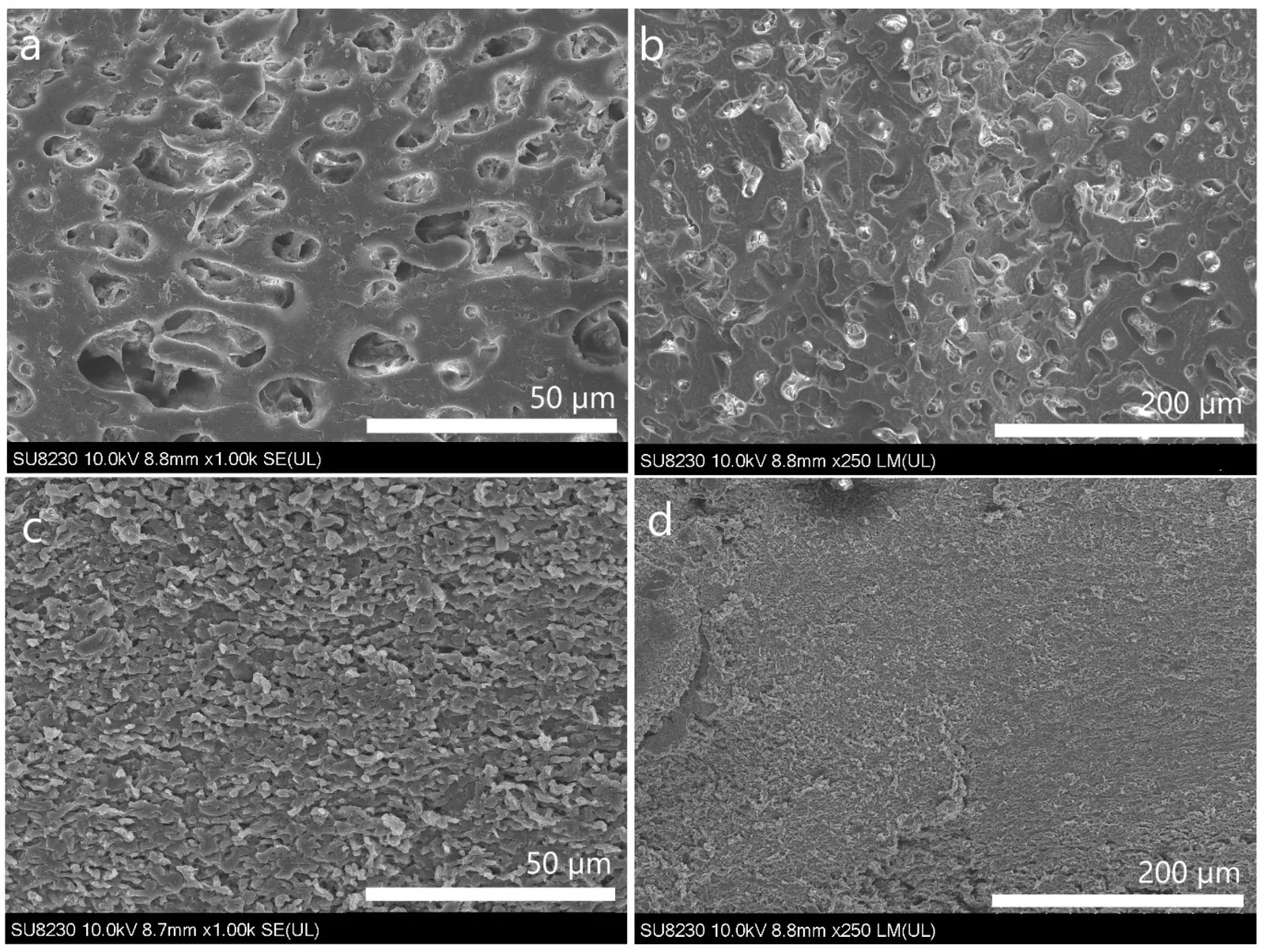
SEM images of PEEK/PEI (**a**,**b**) and PEEK/PEI/CNT 1% (**c**,**d**).

As stated earlier, a PEEK/PEI blend with a 40:60 wt.% composition was employed as the polymer matrix material. This blend was specifically selected to explore the potential of achieving a co-continuous morphology, which could enable the development of a double-percolated structure. To verify the co-continuity, both scanning electron microscopy (SEM) analysis and the selective dissolution method were used. Figure 2 shows the SEM images of the PEEK/PEI and PEEK/PEI/CNT (1 wt.%) blends after selective dissolution of PEI. The images reveal elongated voids, which in fact represent PEI domains, originally dispersed within the PEEK phase. The degree of interconnection between these domains appears sufficient to classify the morphology as co-continuous. To confirm this, the co-continuity of the blend was quantitatively assessed using the selective dissolution method explained in Section 2.4. The degree of continuity for the PEI phase in the PEEK/PEI blend was determined to be 84%, indicating highly interconnected domains. Notably, the sample retained its overall shape after dissolution, suggesting that the remaining PEEK phase also forms a continuous network. These findings confirm that both phases are continuous, and the blend possesses a co-continuous morphology. It is important to note that the addition of CNTs can influence both the morphology and the degree of co-continuity. To assess this effect, the same selective dissolution procedure was applied to several samples investigated in this study, and the resulting continuity values are summarized in Table 6. Based on these results, all samples exhibit co-continuous morphologies and the addition of CNT enhances the degree of continuity. Slight deviations from 100% continuity may arise from the partial swelling or limited solvent absorption of PEEK [19].

**Figure 3:**
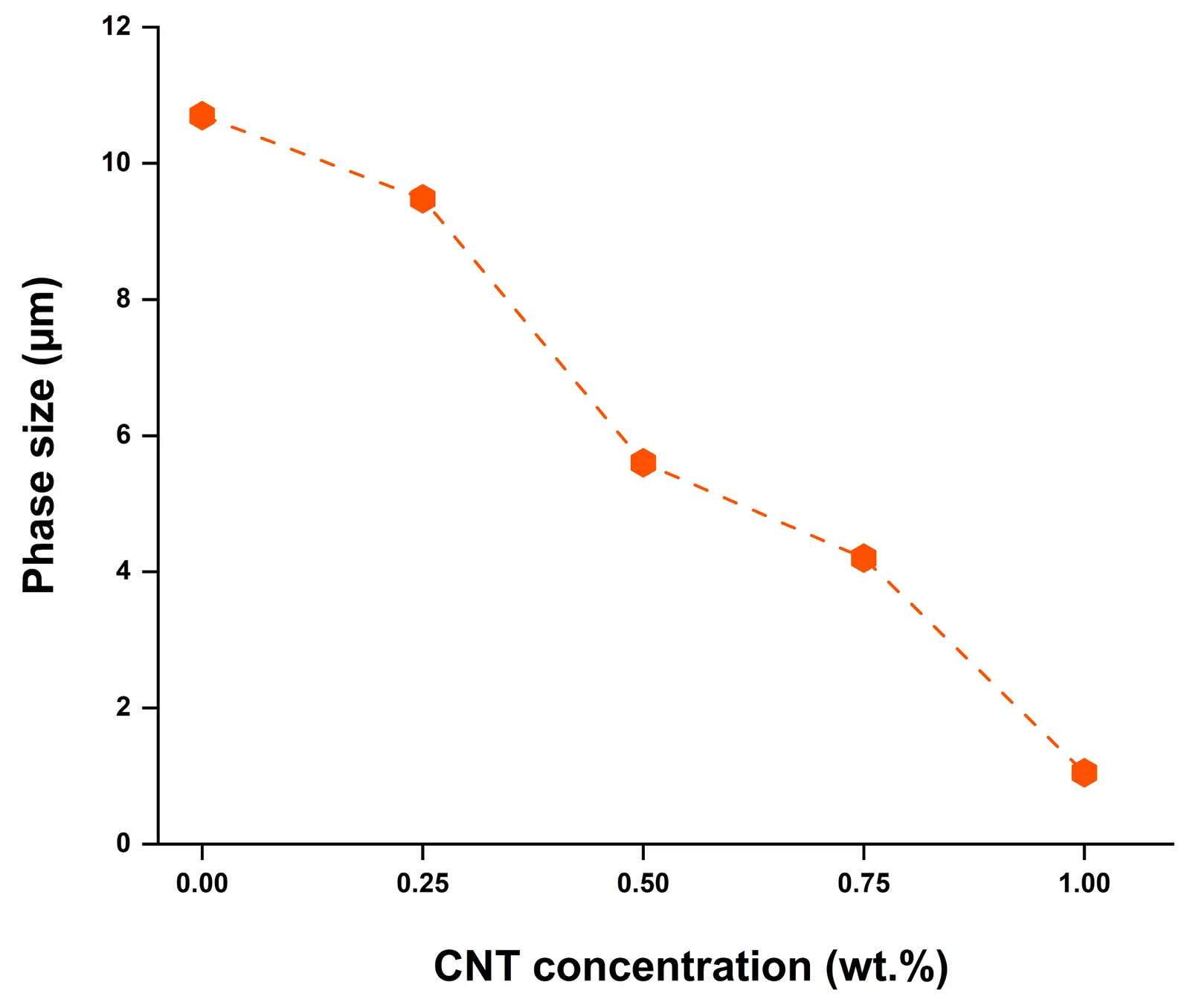
Variations in PEI phase size with CNT concentration in PEEK/PEI/CNT nanocomposites.

The size of the individual domains is another critical parameter that can significantly influence the localization behavior of the filler within a polymer blend. For selective localization, the domains must be sufficiently large to accommodate the filler particles. If the domains are too small, physical confinement may prevent the proper migration or embedding of the nanotubes, potentially disrupting the desired distribution and network formation. Figure 3 illustrates the variation in average domain size as a function of CNT content. The domain size for the neat PEEK/PEI blend is approximately 11 μm and decreases progressively with increasing CNT content, reaching about 1 μm for the sample containing 1 wt.% CNT. As evident, the co-continuous structure is maintained, but the morphology becomes significantly finer compared to the unfilled blend. This refinement of phase morphology is a well-established effect of CNT incorporation [51]. Nevertheless, the domain size remains sufficiently large to allow for selective localization of the nanotubes within one of the phases since nanotubes get broken and become smaller during processing.

### 3.4. Electrical Conductivity and Morphology

#### 3.4.1. Influence of Blending

**Figure 4:**
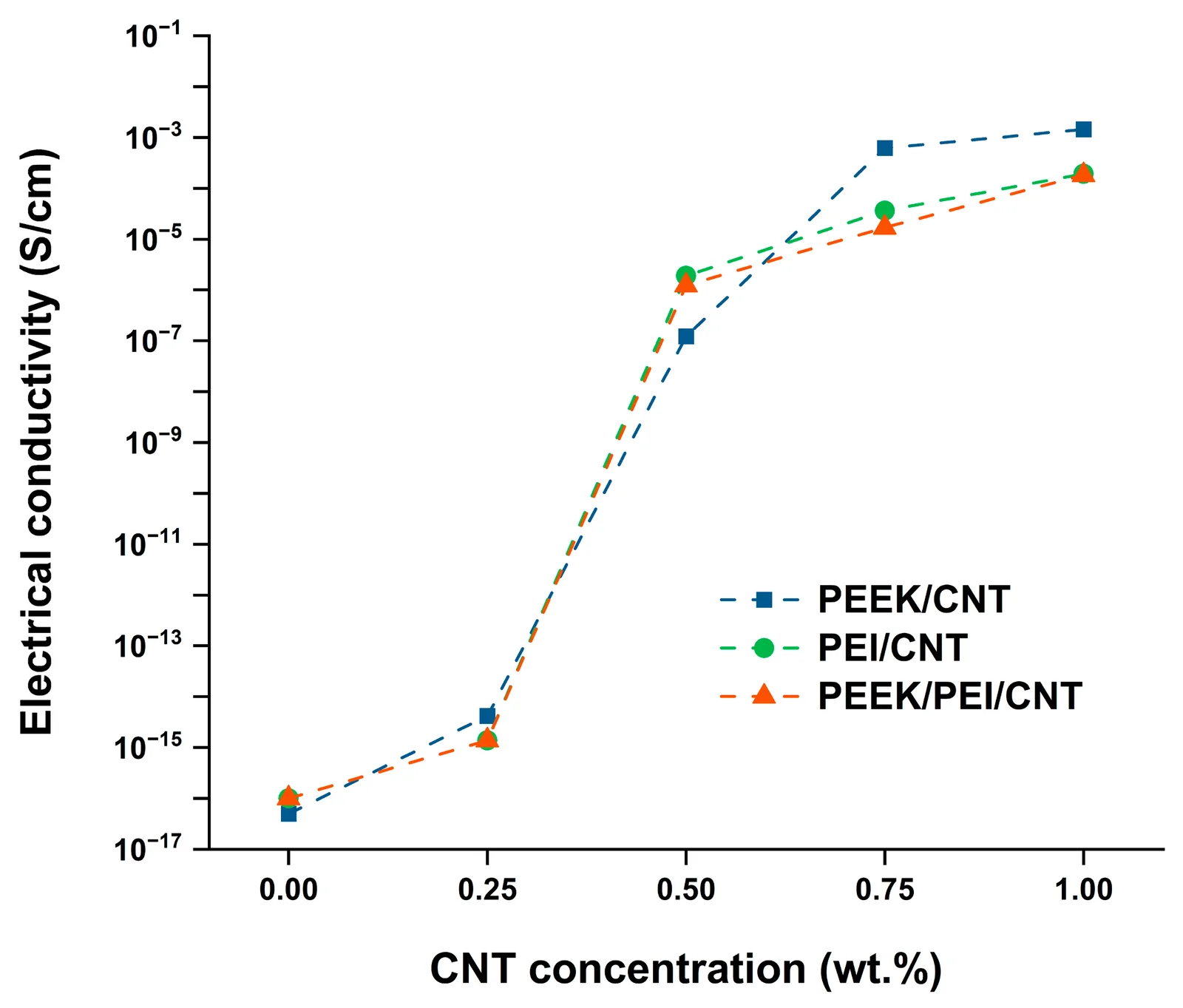
Electrical conductivity of PEEK/CNT, PEI/CNT, and PEEK/PEI/CNT nanocomposites.

Figure 4 illustrates the variation in electrical conductivity of PEEK/CNT, PEI/CNT, and PEEK/PEI/CNT nanocomposites as a function of CNT concentration. The electrical conductivity of nanocomposites could be described by a power-law equation [12,52]:

σ = k(ρ − ρ_c_)^t^

in which σ represents the electrical conductivity of the nanocomposite after percolation, ρ is the mass fraction of nanoparticles, ρ_c_ denotes the percolation threshold, t is a fitting exponent influenced only by the system’s dimensionality, and k is a scaling constant. This model applies specifically to the post-percolation regime, i.e., ρ > ρ_c_ [12,52].

The percolation threshold was estimated by applying linear regression to plots of log(σ) versus log (ρ − ρ_c_) and the obtained values are listed in Table 7. According to the linear regression analysis, the percolation threshold of the PEEK/CNT system is approximately 0.5 wt.%, while those of the PEEK/PEI/CNT and PEI/CNT systems are around 0.3 wt.%. However, since only three data points were available for each series, these values should be considered as ranges rather than exact quantities. Moreover, visual inspection of the conductivity curves indicates that all systems exhibit similar percolation behavior.

The PEI/CNT system is theoretically expected to exhibit a lower percolation threshold than PEEK/CNT due to two main factors: (1) the stronger thermodynamic compatibility of CNTs with PEI, which enhances nanoparticle–polymer interactions and promotes dispersion [42,53,54], and (2) the higher viscosity of PEI, which generates stronger shear forces during mixing, breaking up large CNT agglomerates into smaller, more uniformly distributed clusters [55]. However, as shown in the electrical conductivity plots, the PEEK/CNT and PEI/CNT systems display similar conductivity trends and nearly identical percolation thresholds.

A key factor explaining this observation is the semi-crystalline nature of PEEK. As revealed by DSC results, approximately 36% of the PEEK matrix consists of crystalline domains, which are inaccessible to CNTs [56]. This spatial confinement forces CNTs to localize within the remaining amorphous region, effectively increasing their local concentration and facilitating the formation of conductive networks at lower overall filler content. In contrast, PEI is an amorphous polymer and offers no such exclusion zones, allowing CNTs to distribute uniformly throughout the entire matrix. Consequently, the advantage of PEI’s higher affinity for CNTs and higher viscosity is offset by the lack of this confinement effect, resulting in a percolation threshold similar to that observed in the PEEK/CNT system.

**Figure 5:**
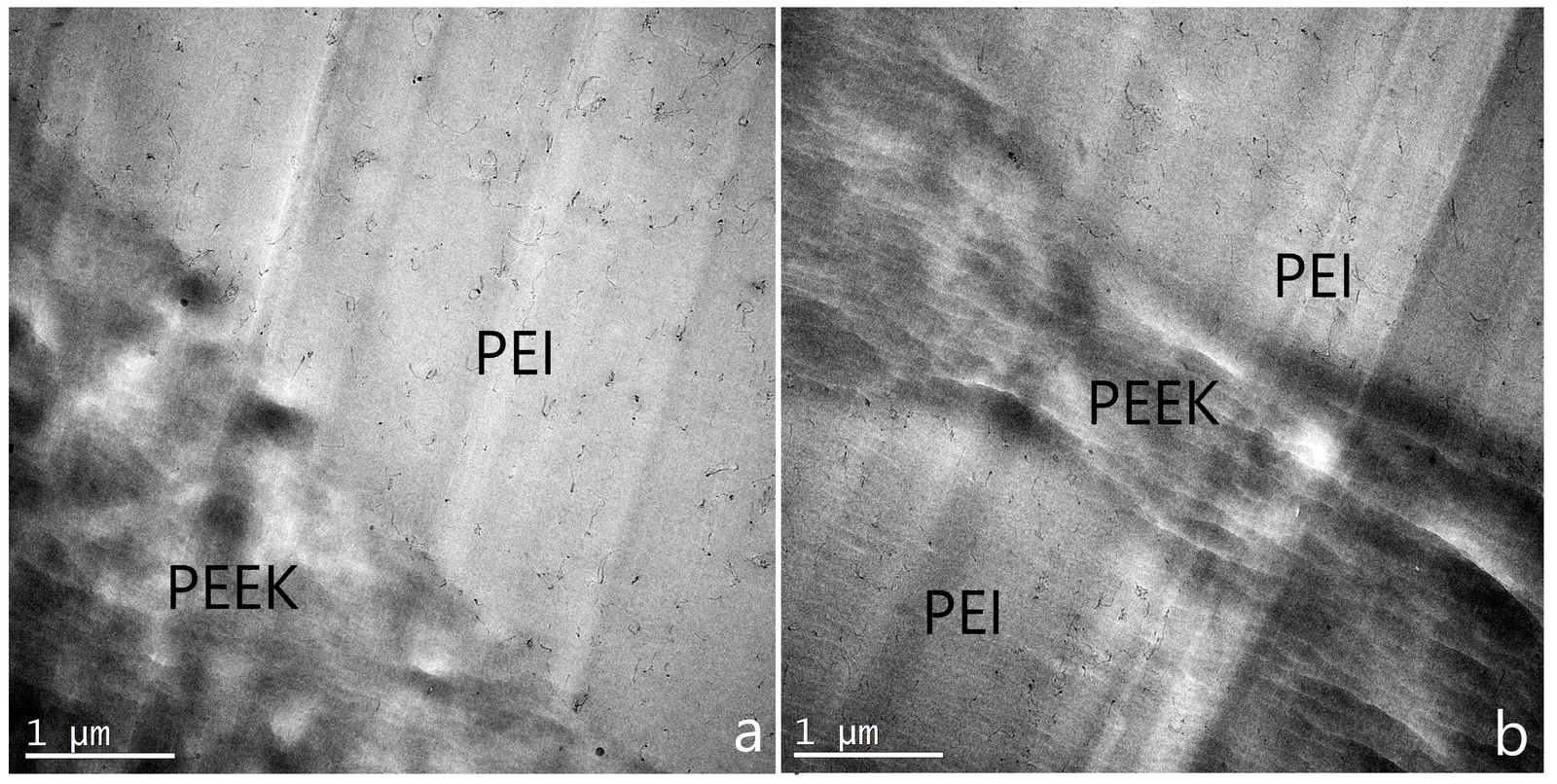
TEM images of PEEK/PEI/CNT 0.25 wt.% (**a**,**b**).

In both the PEEK/CNT and PEEK/PEI/CNT systems, similar electrical percolation behavior is observed with percolation threshold in the range of 0.25–0.5 wt.%. Despite the potential for a double percolation mechanism at the interface or within a specific phase, the PEEK/PEI/CNT nanocomposites do not exhibit a significantly lower percolation threshold compared to PEEK/CNT. This similarity arises because in both systems, a portion of the matrix is excluded and thus not accessible to CNTs. In the case of the PEEK/CNT system, as previously discussed, CNTs are restricted to about 64% of the matrix volume, with the remaining 36% occupied by crystalline regions. In the PEEK/PEI/CNT system, a similar exclusion occurs, though through a different mechanism. Here, 40% of the matrix consists of PEEK and 60% is PEI. As revealed by the TEM images presented in Figure 5, CNTs show a strong preference for the PEI phase, completely migrating away from the PEEK regions. Consequently, CNTs occupy only 60% of the total matrix volume, which explains the comparable percolation thresholds observed in the two systems.

As discussed in Section 3.1, this migration is driven by thermodynamic compatibility and interfacial interactions, which favor CNT localization in the more polar PEI phase. Although the higher viscosity of PEI relative to PEEK could theoretically hinder CNT migration across the phase interface, potentially leading to partial trapping and interfacial network formation [51,57,58], the TEM images confirm complete migration of CNTs into the PEI phase.

**Figure 6:**
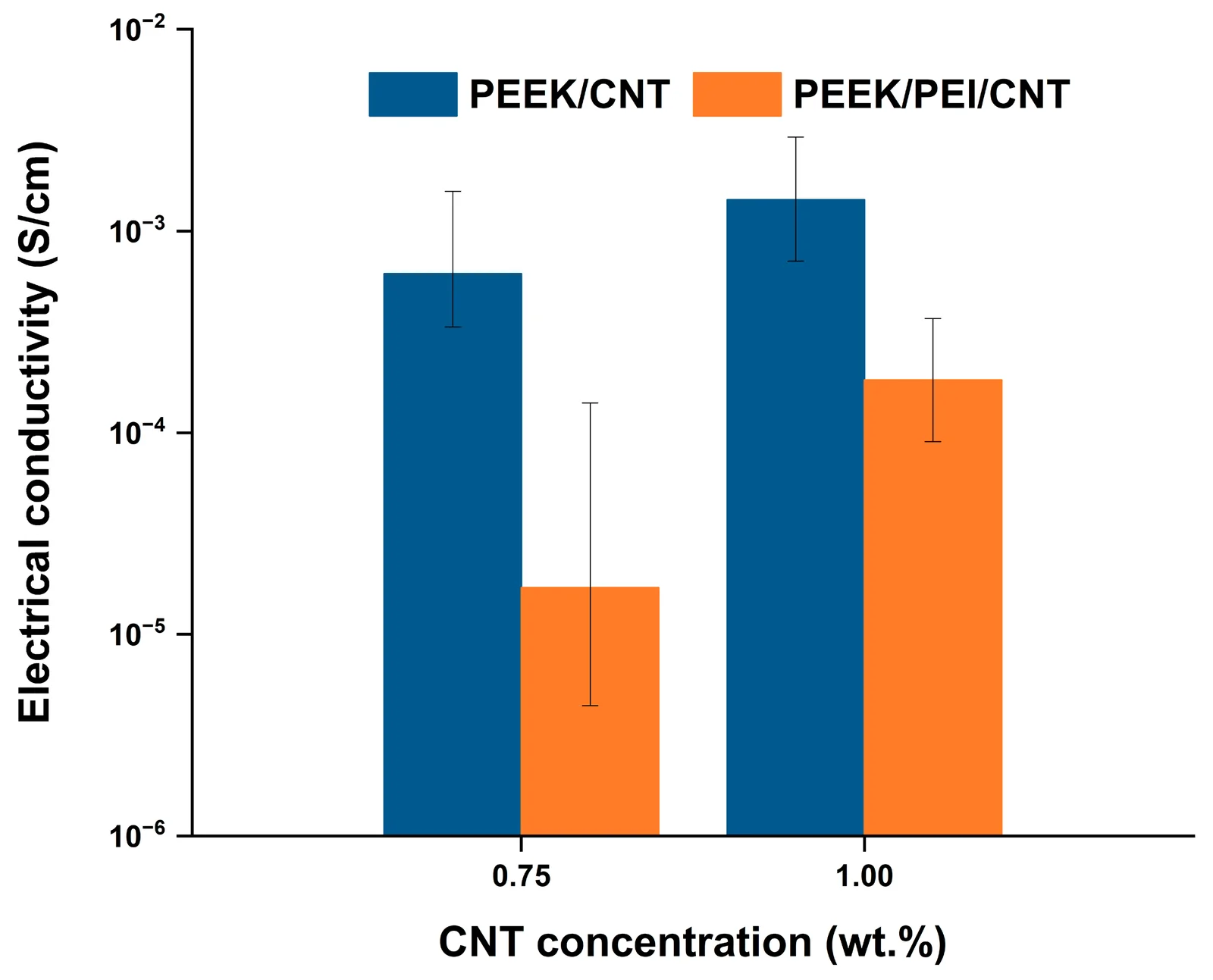
Electrical conductivity of PEEK/CNT and PEEK/PEI/CNT in post percolation regime.

**Figure 7:**
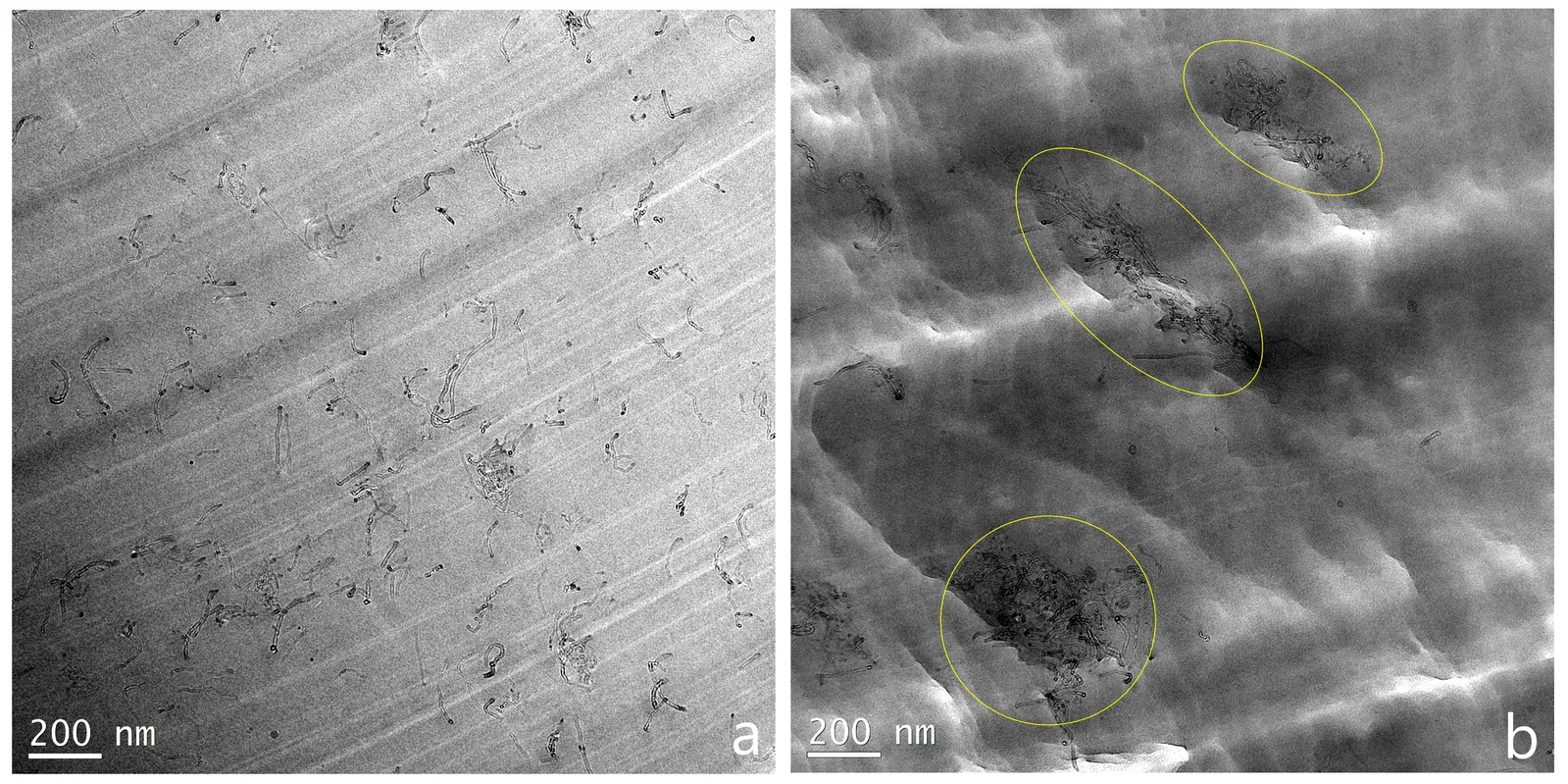
TEM images of PEI/CNT 0.5 wt.% (**a**), and PEEK/CNT 0.5 wt.% (**b**). Yellow circles highlight the presence of nanotube clusters.

Further insights arise from the comparison of conductivity values in the post-percolation regime. As shown in Figure 6, at CNT loadings of 0.75 wt.% and 1 wt.%, the PEEK/CNT nanocomposites exhibit about one order of magnitude higher electrical conductivity than their PEI/CNT counterparts. This difference can be attributed to the quality of CNT dispersion in the respective matrices. In the PEI/CNT system, the stronger wettability of CNTs by the PEI leads to more uniform dispersion and minimal agglomeration, resulting in a homogeneous but less interconnected conductive network. This observation is supported by the TEM images shown in Figure 7. Conversely, the PEEK/CNT samples exhibit noticeable CNT agglomerates (Figure 7). Although these agglomerates delay the percolation, once a conductive network is established, their presence may enhance overall conductivity. The agglomerates act as conductive “hubs” bridging isolated CNT clusters and facilitating electron transport across the matrix [55,59,60,61,62].

The electrical performance obtained in the present work compares favorably with previously reported melt-processed PEEK/CNT nanocomposites. Conventional PEEK/MWCNT systems generally exhibit electrical percolation thresholds between 1 and 4 wt.% CNT, depending on the processing method and nanotube dispersion [4,15,16,17,18]. Gao et al. reduced the percolation threshold to approximately 0.6 wt.% by employing a co-continuous PEEK/TPI blend with selective MWCNT localization, whereas their binary PEEK/CNT system exhibited a percolation threshold of approximately 1.3 wt.% [24,25,26]. In contrast, both the PEEK/CNT and PEEK/PEI/CNT nanocomposites investigated in the present study exhibit lower percolation thresholds of approximately 0.25–0.5 wt.%. Although the maximum electrical conductivity is slightly lower than that reported for systems containing higher CNT loadings, comparable conductive performance was achieved using only 1 wt.% CNT. These results demonstrate that the spatial confinement mechanisms investigated in this work are effective in reducing the amount of conductive filler required to achieve electrical percolation.

#### 3.4.2. Rheological Percolation Threshold

**Figure 8:**
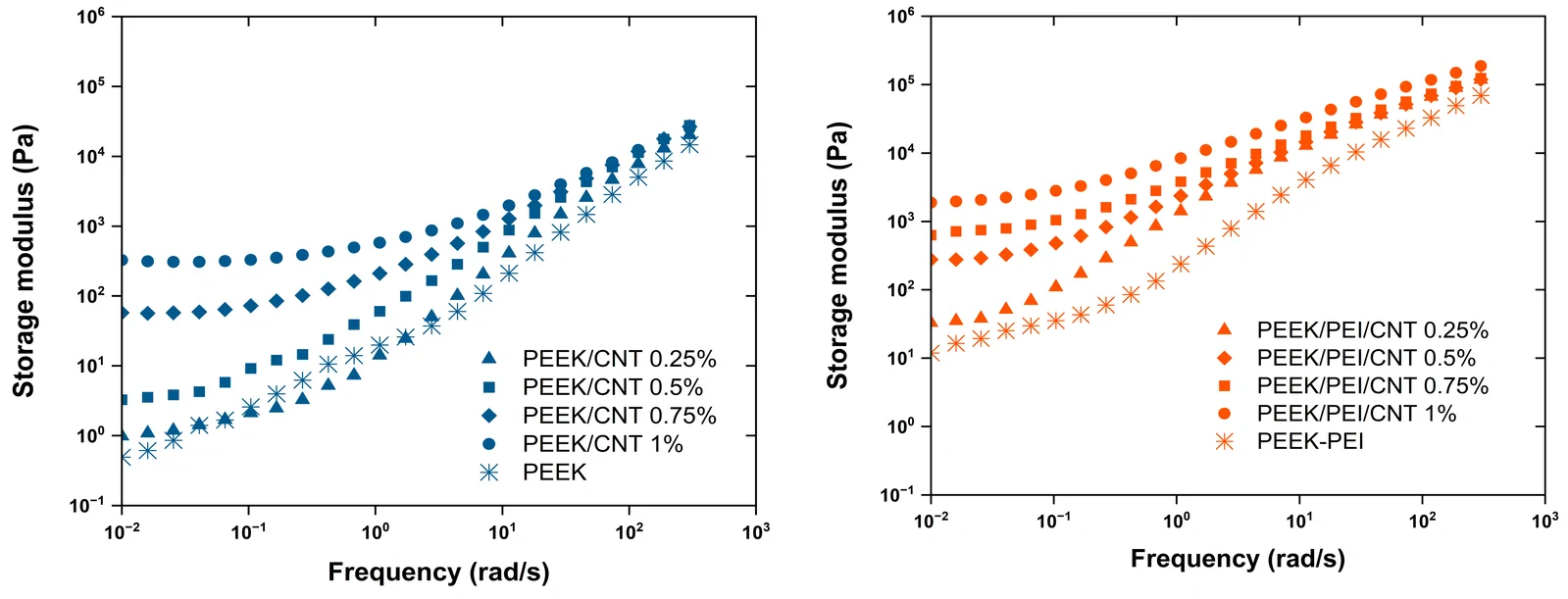
Storage modulus vs frequency of PEEK/CNT and PEEK/PEI/CNT nanocomposite system.

Figure 8 presents the variation in storage modulus as a function of angular frequency for PEEK/CNT and PEEK/PEI/CNT samples. For both systems, all samples exhibit a plateau in the low-frequency region, where the modulus becomes nearly independent of frequency. This behavior indicates the formation of a continuous CNT network that constrains polymer chain mobility and imparts solid-like viscoelastic properties of the nanocomposites [63,64,65].

The concept of the percolation threshold extends beyond electrical conductivity and is equally relevant to rheological behavior. When the concentration of nanoparticles exceeds a critical level, the system undergoes a rheological transition associated with the formation of a continuous network within the polymer matrix. Below the percolation threshold, nanoparticles remain isolated and do not engage in particle–particle or short-range interactions; instead, their interactions are primarily with the surrounding matrix. However, once the percolation threshold is surpassed, the nanoparticles form a mesoscale randomly connected network. This structure introduces long-range interactions between particles, restricts polymer chain mobility and significantly alters the material’s rheological properties [63,64].

**Figure 9:**
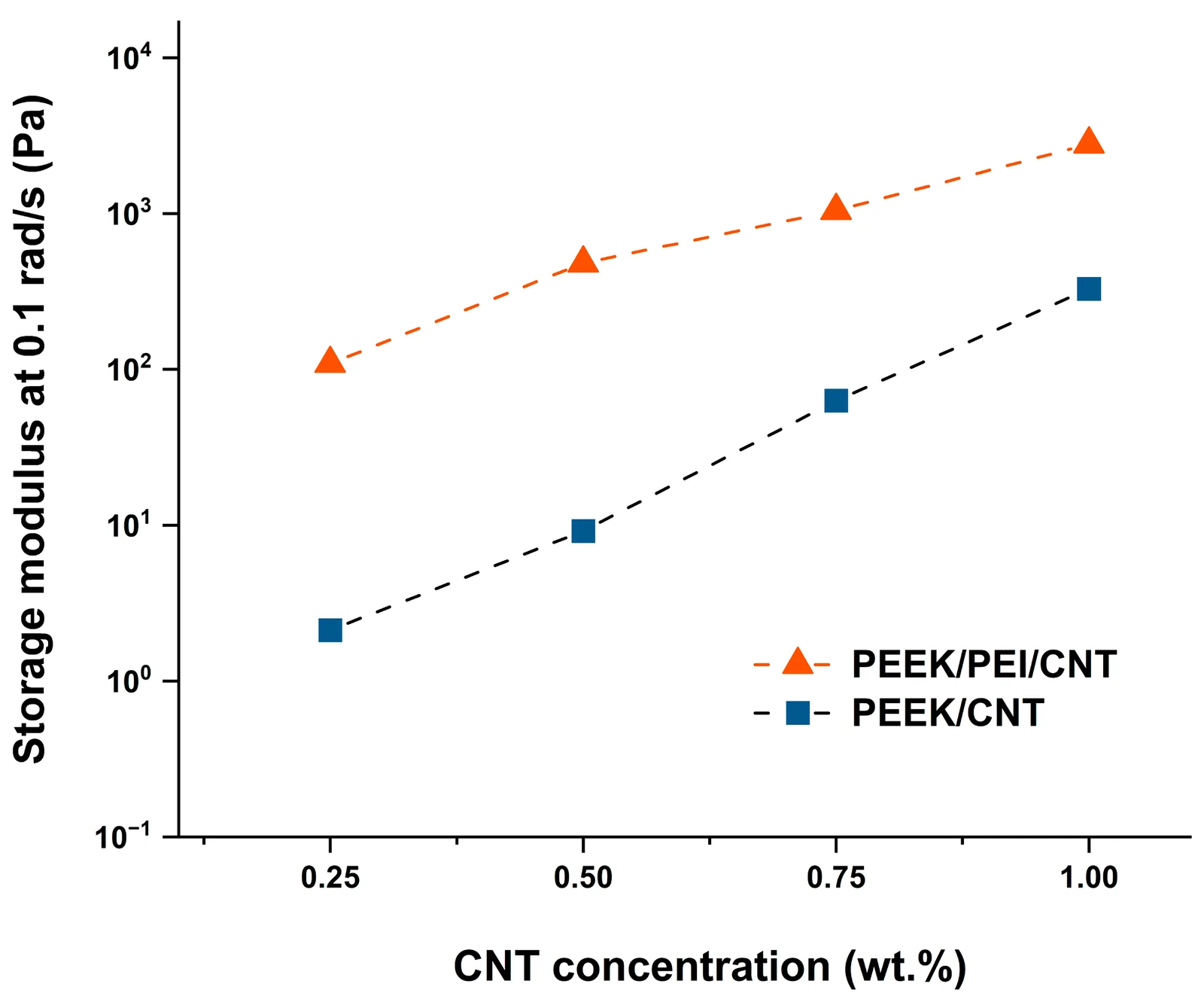
Storage modulus measured at a frequency of 0.1 rad/s for PEEK/CNT and PEEK/PEI/CNT nanocomposite systems.

To identify the rheological percolation threshold, the value of storage modulus at a specific low frequency (0.1 rad/s) was plotted vs CNT content (Figure 9). In both series of samples, the storage modulus demonstrates a relatively linear and gradual increase as a function of CNT content. This behavior suggests that the rheological percolation threshold is achieved at a concentration below 0.25 wt.%, placing the system in a post-percolation regime. In this state, a connected CNT network is already established, and further increases in CNT concentration primarily enhance the network’s strength rather than initiate its formation. This finding aligns with previous studies reporting that the rheological percolation generally occurs at lower CNT concentrations than electrical percolation, as mechanical connectivity requires fewer and less continuous contacts than those needed to establish an electrically conductive path [64,66].

#### 3.4.3. Influence of Mixing Time

**Figure 10:**
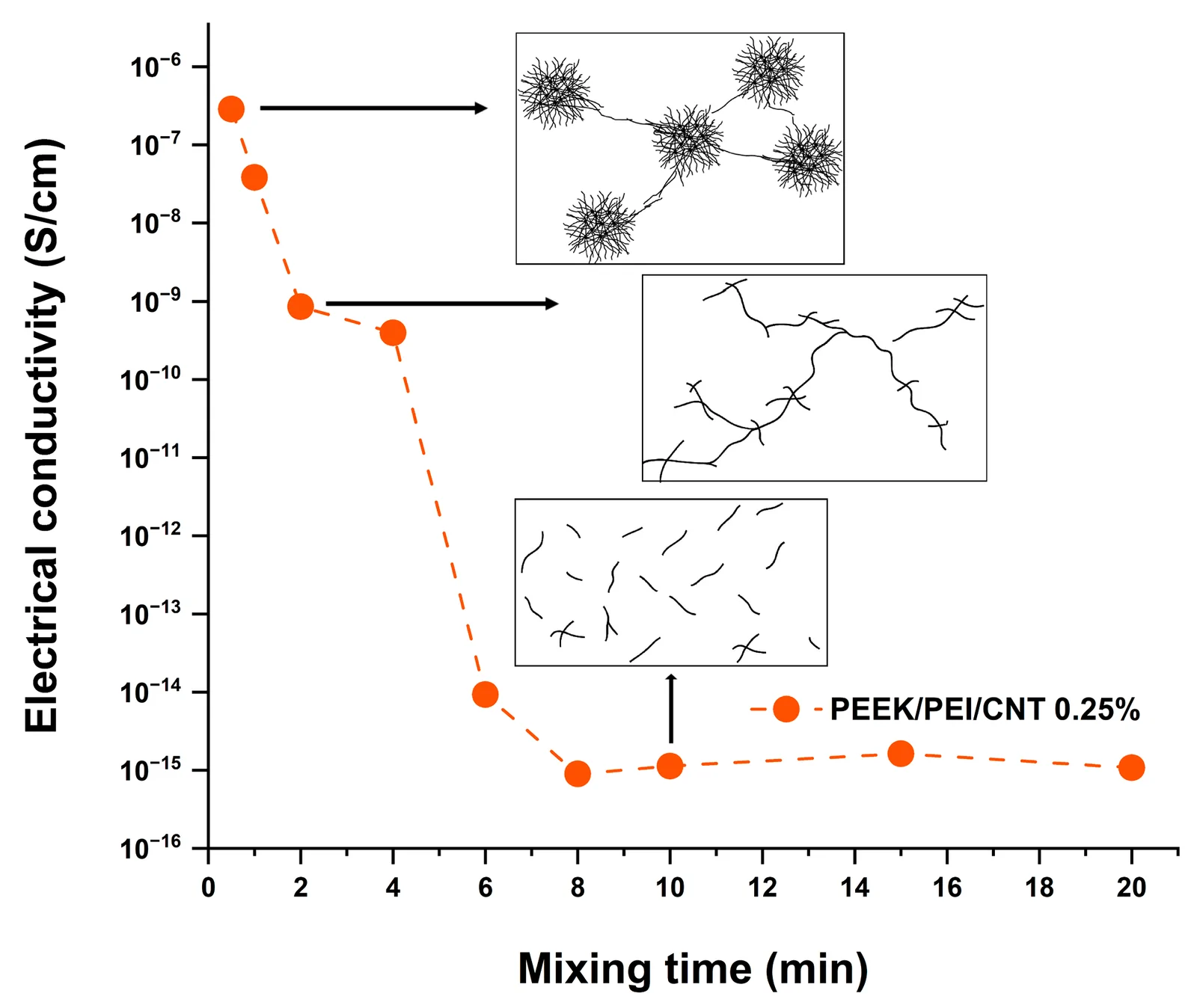
Variations in electrical conductivity of PEEK/PEI/CNT 0.25 wt.% sample with processing time.

As shown by the TEM images of the PEEK-PEI-CNT samples discussed previously, all nanotubes are located within the PEI phase, despite being initially dispersed in the PEEK phase at the start of mixing. This observation indicates that during melt mixing, CNTs migrate from the PEEK to the PEI phase. All the samples discussed so far were prepared using a 10 min melt mixing process. To better understand the migration dynamics of CNTs during the mixing process, the influence of mixing time on the filler localization and electrical conductivity was investigated. For this purpose, the PEEK/PEI/CNT nanocomposites containing 0.25 wt.% CNT were prepared with mixing times ranging from 30 s to 20 min. The electrical conductivity of each sample was measured and the results are presented in Figure 10. These data provide insight into the temporal evolution of CNT arrangement and localization, as reflected by changes in conductivity and schematic images of the CNT arrangement in the figure.

The sample mixed for 30 s exhibits the highest electrical conductivity, indicating the presence of a well-connected CNT network. As mixing time increases, conductivity gradually decreases, and after sufficiently prolonged mixing (6–20 min), the nanocomposite becomes non-conductive, reflecting the loss of a continuous conductive pathway within the polymer matrix. This trend suggests that double percolation does not occur at the interphase, or, if it does, it occurs only within the very first seconds of mixing. Otherwise, a transient peak in conductivity would be expected at intermediate mixing times due to the temporary accumulation of nanotubes at the interphase [67,68,69]. The absence of such behavior can be attributed to the low interfacial tension between PEEK and PEI, which imposes little thermodynamic resistance to nanoparticle migration [70]. In addition, the significantly higher viscosity of PEI compared to PEEK does not appear to provide a sufficient kinetic barrier to hinder CNT migration.

**Figure 11:**
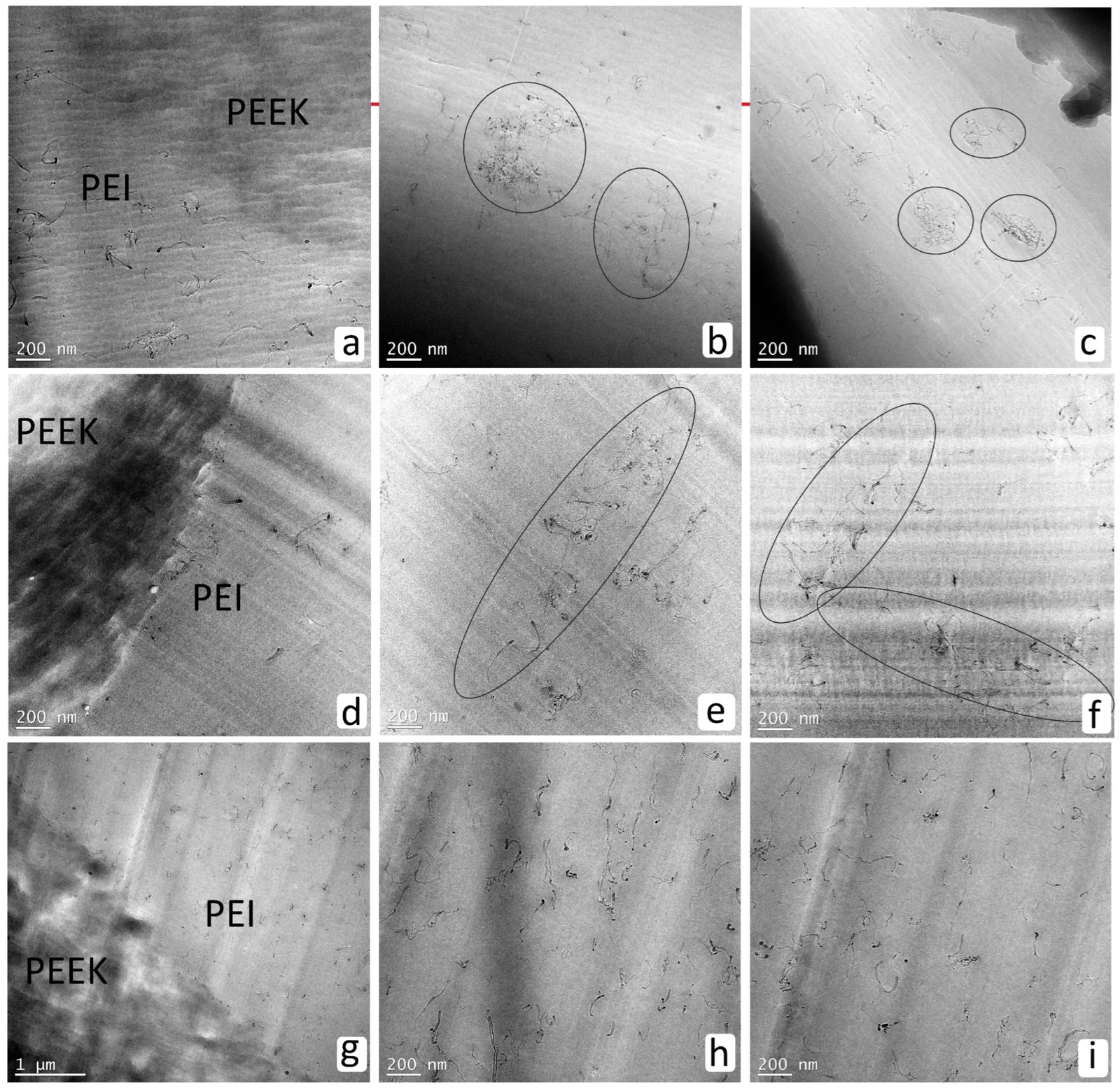
TEM images of PEEK/PEI/CNT 0.25 wt.% at different mixing times: 30 s (**a**–**c**), 2 min (**d**–**f**), 10 min (**g**–**i**). Black circles highlight the presence of nanotube clusters.

TEM images of different mixing stages (30 s, 2 min, and 10 min) shown in Figure 11 corroborate these findings. In all cases, at the beginning of the mixing stage, CNTs are fully located within the PEI phase, confirming the rapid migration of nanotubes during melt mixing. However, their morphology evolves markedly with time. After 30 s, large CNT agglomerates—originally formed in the PEEK phase—are still visible within the PEI phase, creating conductive interconnections that promote high electrical conductivity. After 2 min, TEM observations reveal partial disintegration of agglomerates due to the high shear forces imposed by the viscous PEI matrix. As a result, conductivity decreases, although the remaining CNT network is still sufficient to sustain electron transport. By 10 min of mixing, the CNTs are well-dispersed throughout the matrix, but their interconnectivity is lost, resulting in the disappearance of the conductive network and the transition to insulating behavior. These findings from TEM images are also depicted schematically in Figure 10.

Overall, these results demonstrate that shorter mixing times are more favorable for achieving higher electrical conductivity and a lower percolation threshold in the PEEK/p-PEI/CNT nanocomposite system used in the present study. This highlights the potential of continuous extrusion as a practical and widely used technique for producing the structural components of the thermoplastic lunar rover.

#### 3.4.4. Influence of Mixing Sequence

**Figure 12:**
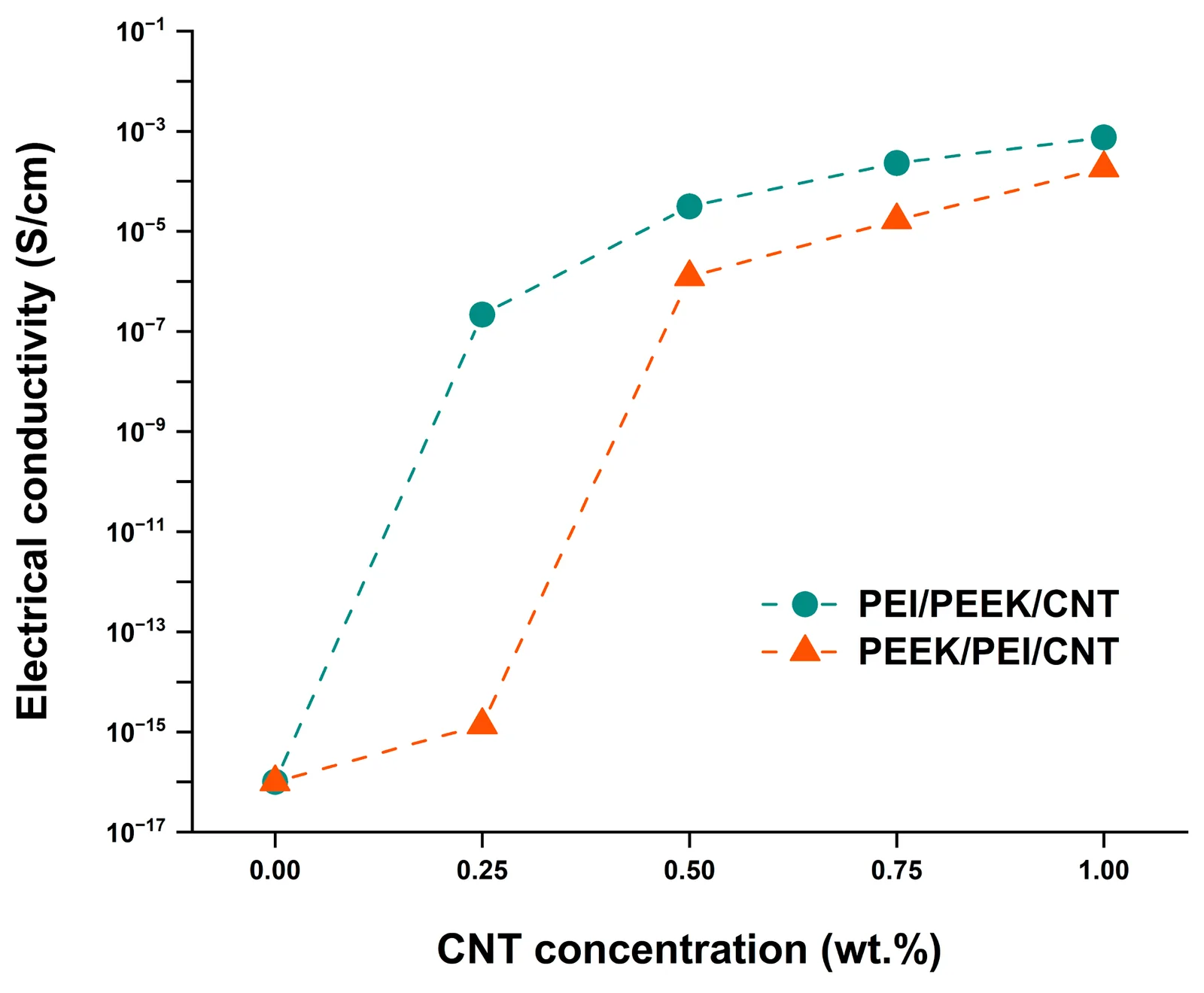
Variations in electrical conductivity of PEEK/PEI/CNT and PEI/PEEK/CNT with CNT concentration.

Figure 12 illustrates the variation in electrical conductivity as a function of CNT content for two different systems: PEEK/PEI/CNT and PEI/PEEK/CNT. The key distinction between these systems lies in their preparation method. In the PEEK/PEI/CNT samples, a PEEK/CNT masterbatch was first prepared, meaning that CNTs were initially dispersed in the PEEK matrix and then blended with PEI. Conversely, in the PEI/PEEK/CNT samples, CNTs were first mixed with PEI before being blended with PEEK.

**Figure 13:**
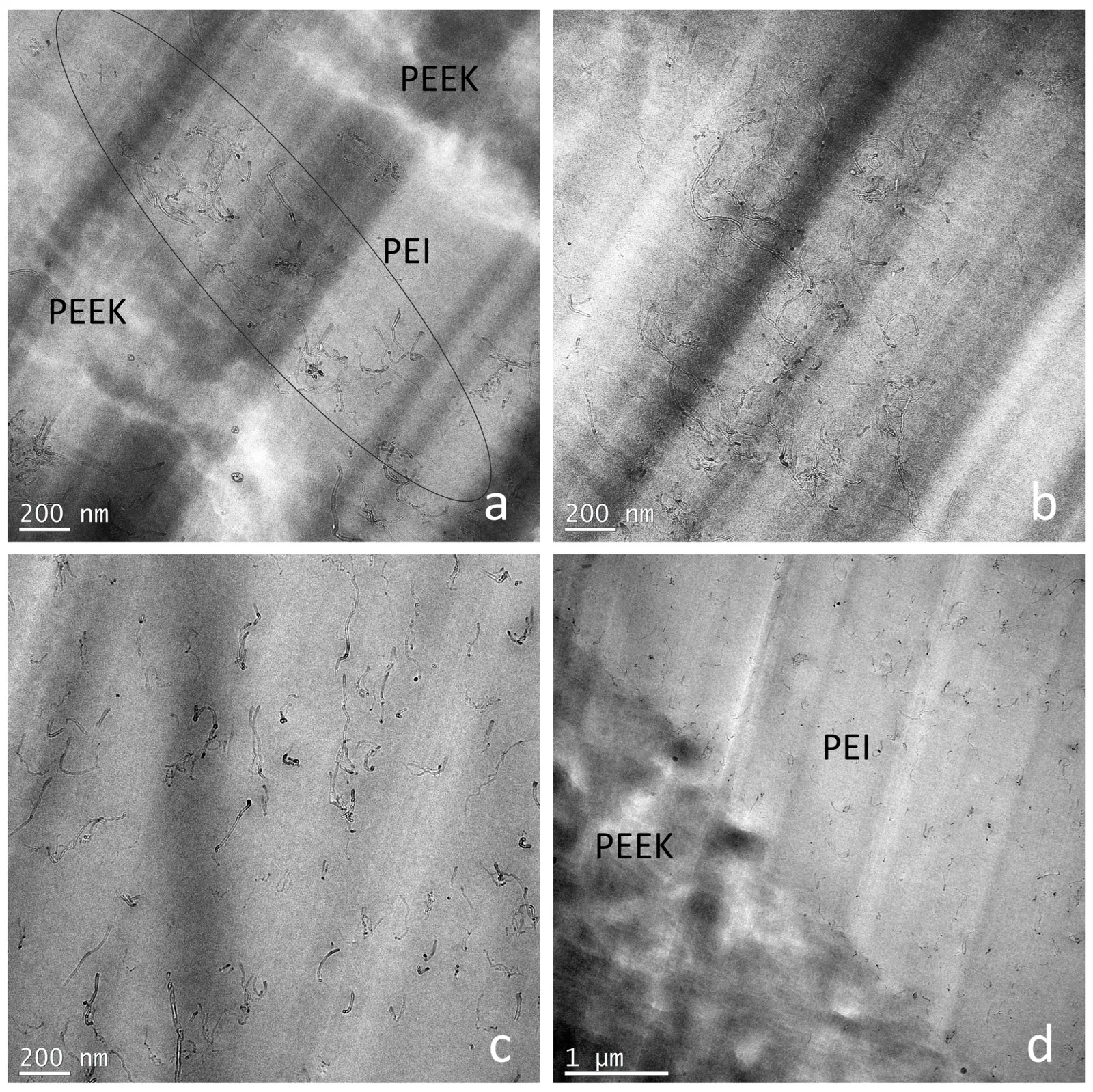
TEM images of PEI/PEEK/CNT 0.25 wt.% (**a**,**b**), and PEEK/PEI/CNT 0.25 wt.% (**c**,**d**). The black circle highlights elongated nanotube clusters.

As observed in the figure, the electrical percolation threshold for the PEI/PEEK/CNT system is below 0.25 wt.%, which is lower than that of the PEEK/PEI/CNT system (0.25–0.5 wt.%)**.** This difference may seem unexpected, given that in both sample series, the CNT localization is the same. In fact, in the PEI/PEEK/CNT system, the strong affinity of CNTs for PEI causes nearly all particles to remain in that phase. On the other side, in the PEEK/PEI/CNT samples, as previously noted, the CNTs all migrate into the PEI phase after prolonged mixing. Thus, despite the different pathways, in both series, the CNTs are finally located in the PEI phase. However, as shown in the TEM images in Figure 13, the microstructure of the CNT network differs notably between the two systems. In the PEI/PEEK/CNT samples, CNTs form interconnected pathways that are essential for efficient electron transport and a reduced percolation threshold. By contrast, the PEEK/PEI/CNT samples contain well-dispersed CNTs that remain isolated and fail to establish continuous conductive pathways. Although this dispersion appears uniform, it is not conducive to high conductivity or a low percolation threshold.

The difference between the two systems can be explained by their processing history. When a PEI/CNT masterbatch is prepared, CNTs are first dispersed directly in the PEI phase, where they can already form a connected network. During subsequent blending with PEEK, this network is largely preserved or easily re-established within the PEI domains, leading to continuous conductive pathways and a lower percolation threshold. In contrast, when a PEEK/CNT masterbatch is used, CNTs are initially incorporated into PEEK as agglomerates. During blending with PEI, CNTs migrate into the PEI phase, where the significantly higher shear forces cause the agglomerates to shatter. The nanotubes are rapidly dispersed into smaller, well-separated units upon entering the PEI matrix, rather than maintaining their initial clustered form. Although they ultimately localize in PEI, their spatial isolation hinders the formation of a continuous conductive network.

#### 3.4.5. Influence of Thermal Annealing

Another process that can significantly influence the localization of the carbon nanotubes and their arrangement in the polymer matrix is thermal annealing [12,13,59,71]. Thermal annealing can alter microstructure by promoting CNT redistribution and improving interconnectivity [12,13,55], or, conversely, by intensifying agglomeration depending on the interfacial interactions and processing conditions [72]. In this context, dynamic percolation describes the evolving nature of the CNT network during annealing, where the electrical conductivity changes over time due to filler migration and rearrangement, relaxation of polymer chains, and morphological adjustments within the blend [13,59,71].

**Figure 14:**
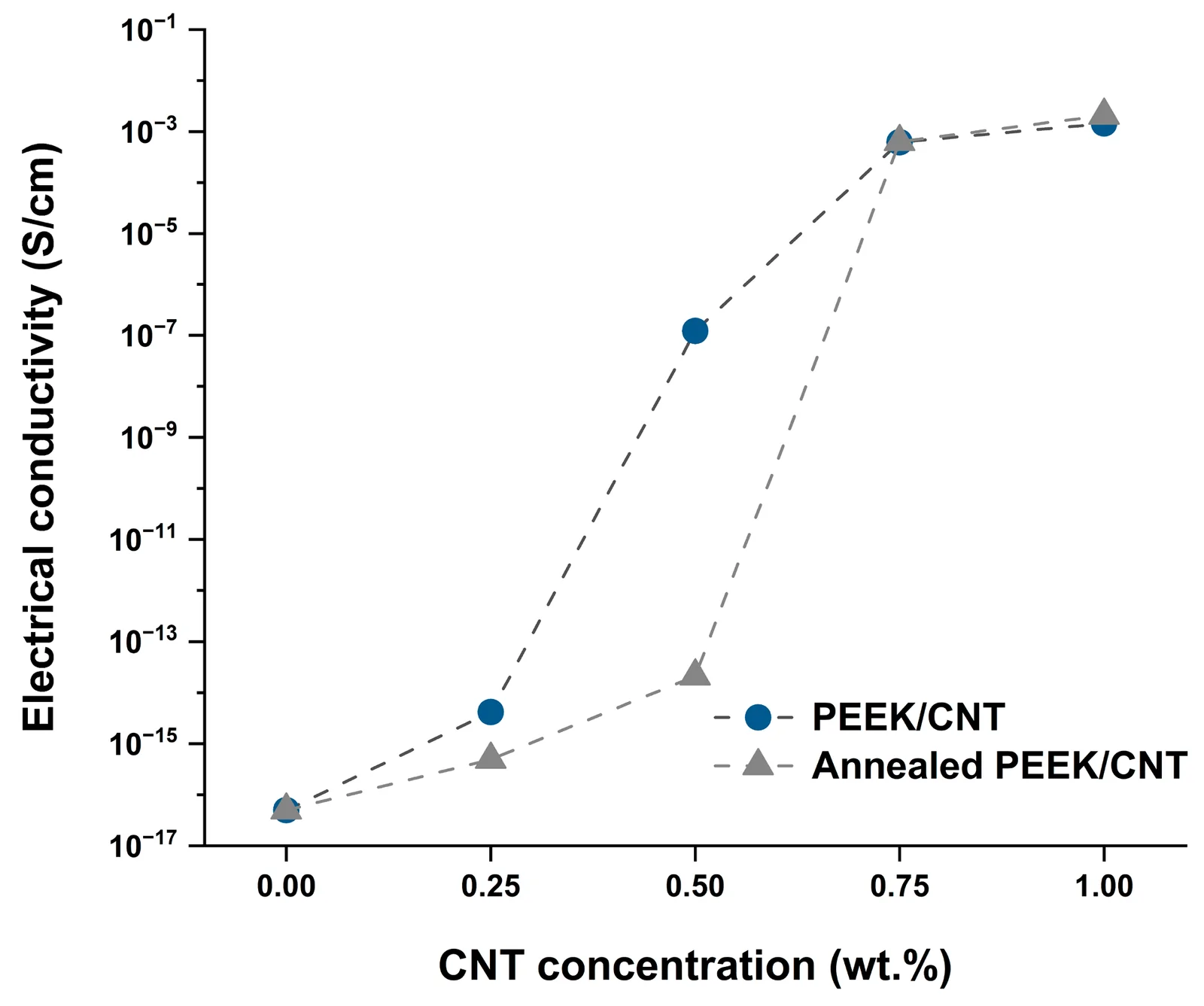
Variations in electrical conductivity of PEEK/CNT and annealed PEEK/CNT with CNT concentration.

Figure 14 presents the electrical conductivity of PEEK/CNT nanocomposites before and after thermal annealing. Notably, the sample containing 0.5 wt.% CNT experiences a significant reduction in conductivity following annealing, corresponding to an increase in the percolation threshold—from 0.25–0.50 wt.% before annealing to 0.50–0.75 wt.% after annealing. This change indicates that the CNT network, initially continuous at 0.5 wt.%, becomes disconnected after annealing. In other words, the interconnections between individual CNTs weaken to an extent that a system-spanning network can no longer form at this concentration.

**Figure 15:**
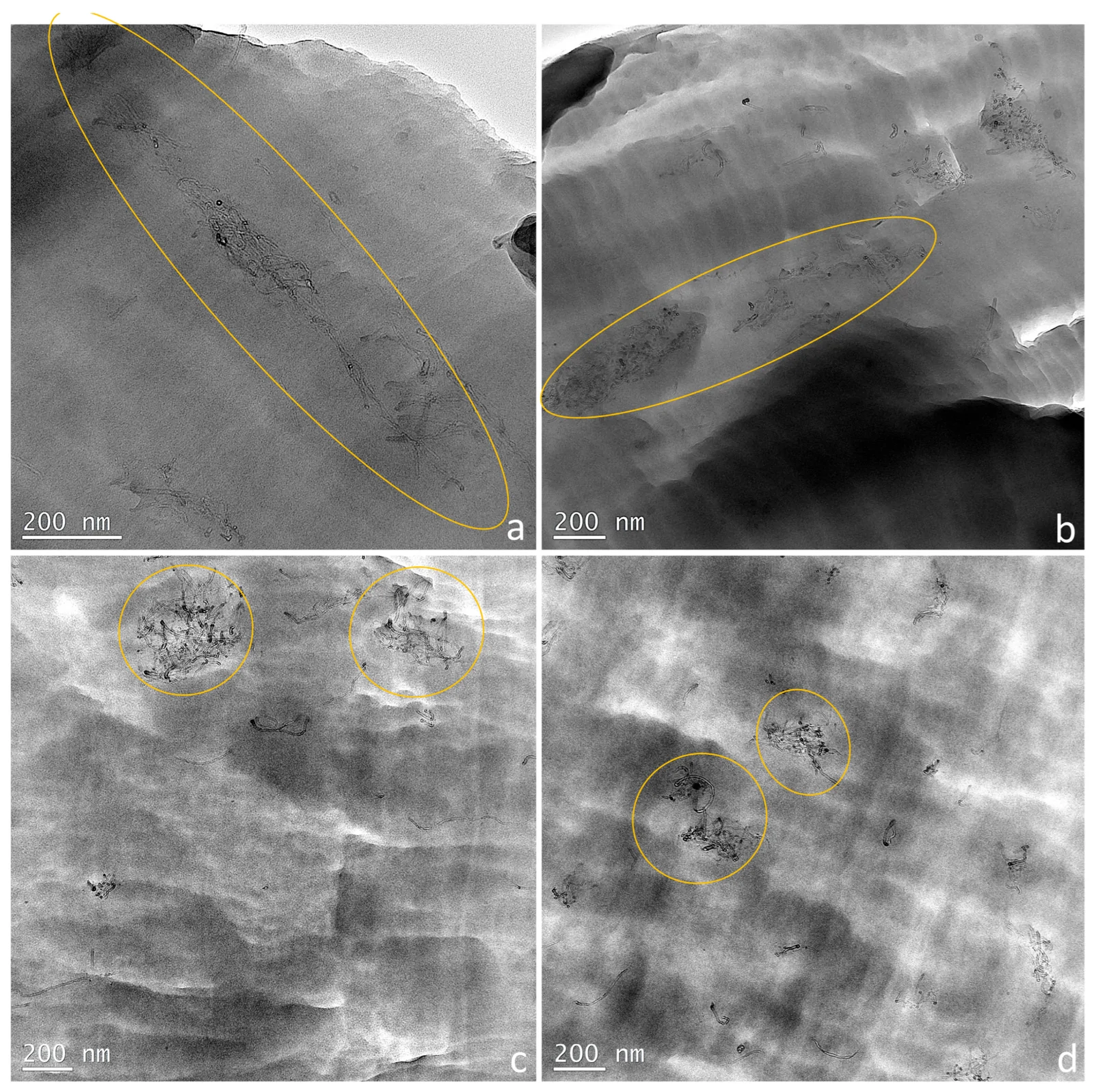
TEM images of PEEK/CNT 0.5 wt.% (**a**,**b**), and annealed PEEK/CNT 0.5 wt.% (**c**,**d**). The yellow circles highlight the presence of nanotube clusters.

TEM images of the PEEK/CNT 0.5 wt.% sample before and after annealing (Figure 15) further substantiate this interpretation. The unannealed sample shows elongated CNT clusters with visible interconnectivity, while the annealed sample reveals larger, more spatially separated CNT agglomerates. The increased size and separation of these clusters suggest that annealing promotes CNT reorganization through thermally activated reaggregation. This behavior arises from the inherent tendency of CNTs to form clusters due to van der Waals forces, thereby reducing interfacial energy [72,73]. During annealing, the system gains sufficient thermal energy to overcome kinetic barriers, allowing CNTs to move and re-aggregate into larger clusters. While this thermodynamically driven process may reduce overall system energy, it disrupts the previously formed conductive network by increasing inter-particle distances, particularly at lower CNT loadings. As a result, conductivity decreases and the percolation threshold shifts to a higher CNT concentration [72].

**Figure 16:**
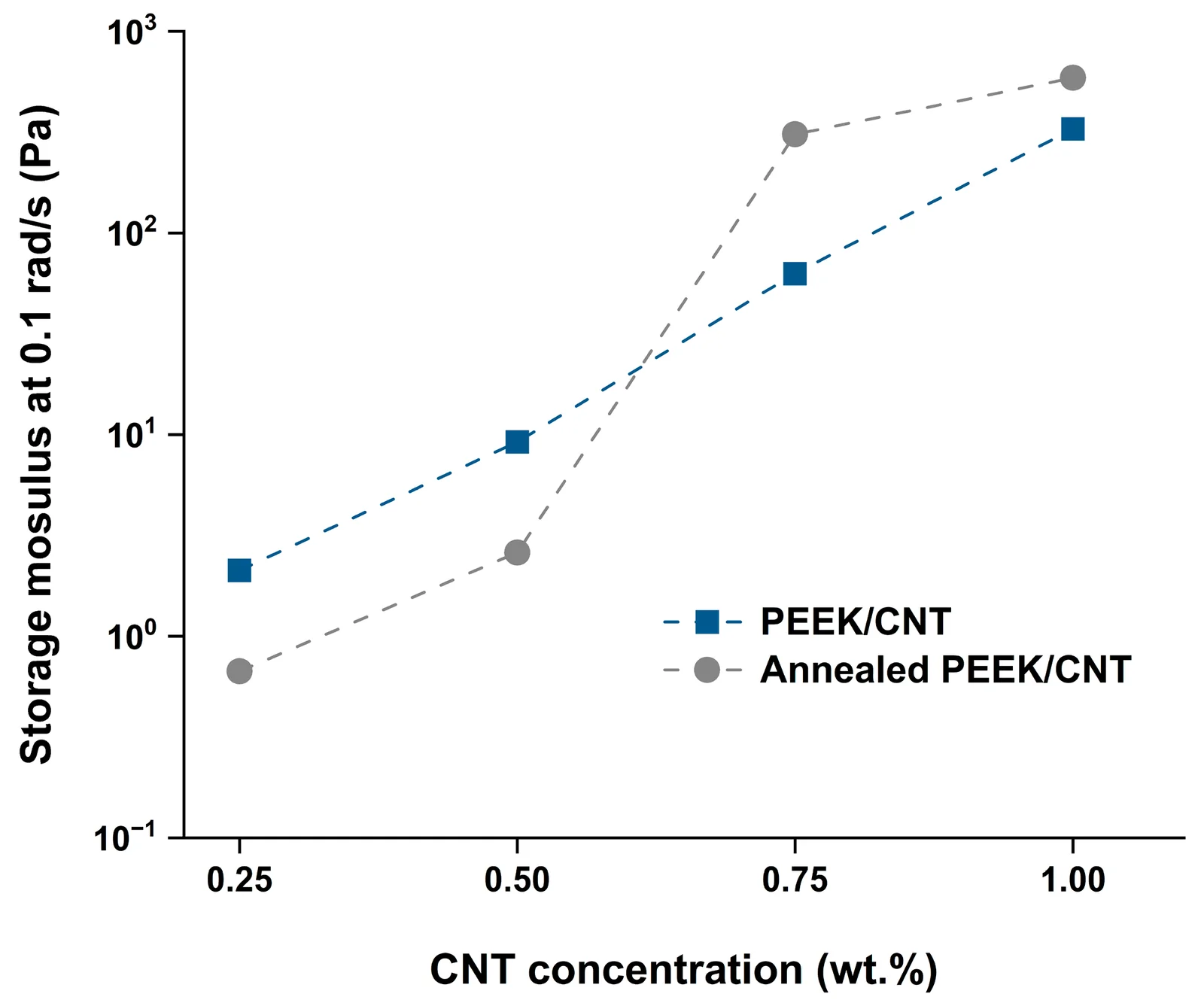
Storage modulus measured at a frequency of 0.1 rad/s for PEEK/CNT and annealed PEEK/CNT.

The rheological data also support this conclusion. Figure 16 presents the variations in storage modulus measured at a frequency of 0.1 rad/s for PEEK/CNT nanocomposites, both before and after thermal annealing. In the case of the unannealed PEEK/CNT samples, the storage modulus demonstrates a relatively linear and gradual increase as a function of CNT content. This behavior suggests that the rheological percolation threshold is achieved at a concentration below 0.25 wt.%. With further addition of CNTs, this pre-existing network is reinforced, leading to a higher storage modulus.

In contrast, the annealed PEEK/CNT samples exhibit a distinct and more dramatic increase in storage modulus between 0.25 wt.% and 0.50 wt.% of nanotubes. This pronounced upturn indicates a delayed onset of rheological percolation compared to the unannealed samples. The shift in the rheological percolation threshold toward higher CNT concentrations after thermal annealing is consistent with the electrical conductivity and TEM images, confirming that annealing promotes the formation of larger, more isolated agglomerates, thereby delaying network development.

**Figure 17:**
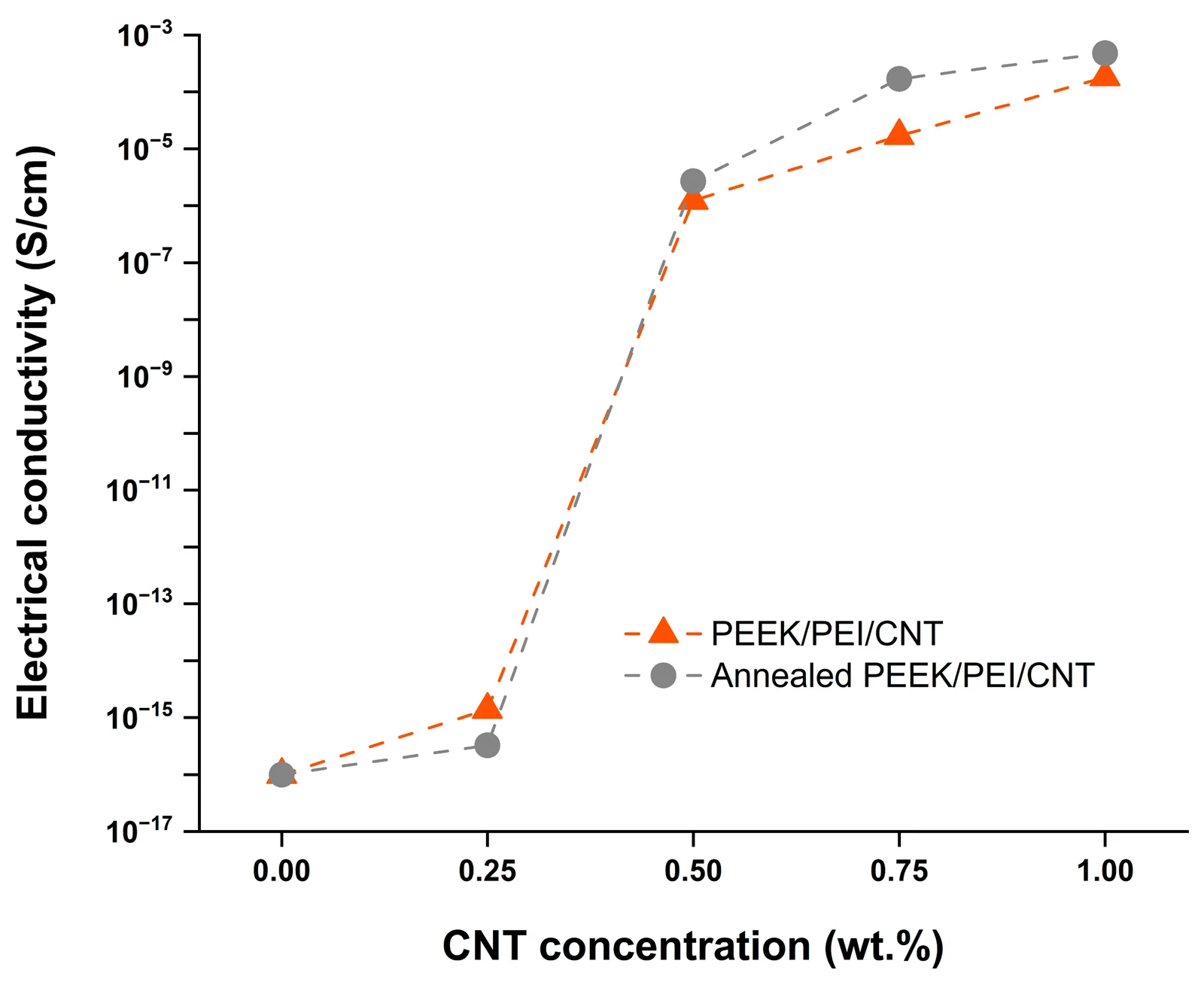
Variations in electrical conductivity of PEEK/CNT and PEEK/PEI/CNT with CNT concentration.

**Figure 18:**
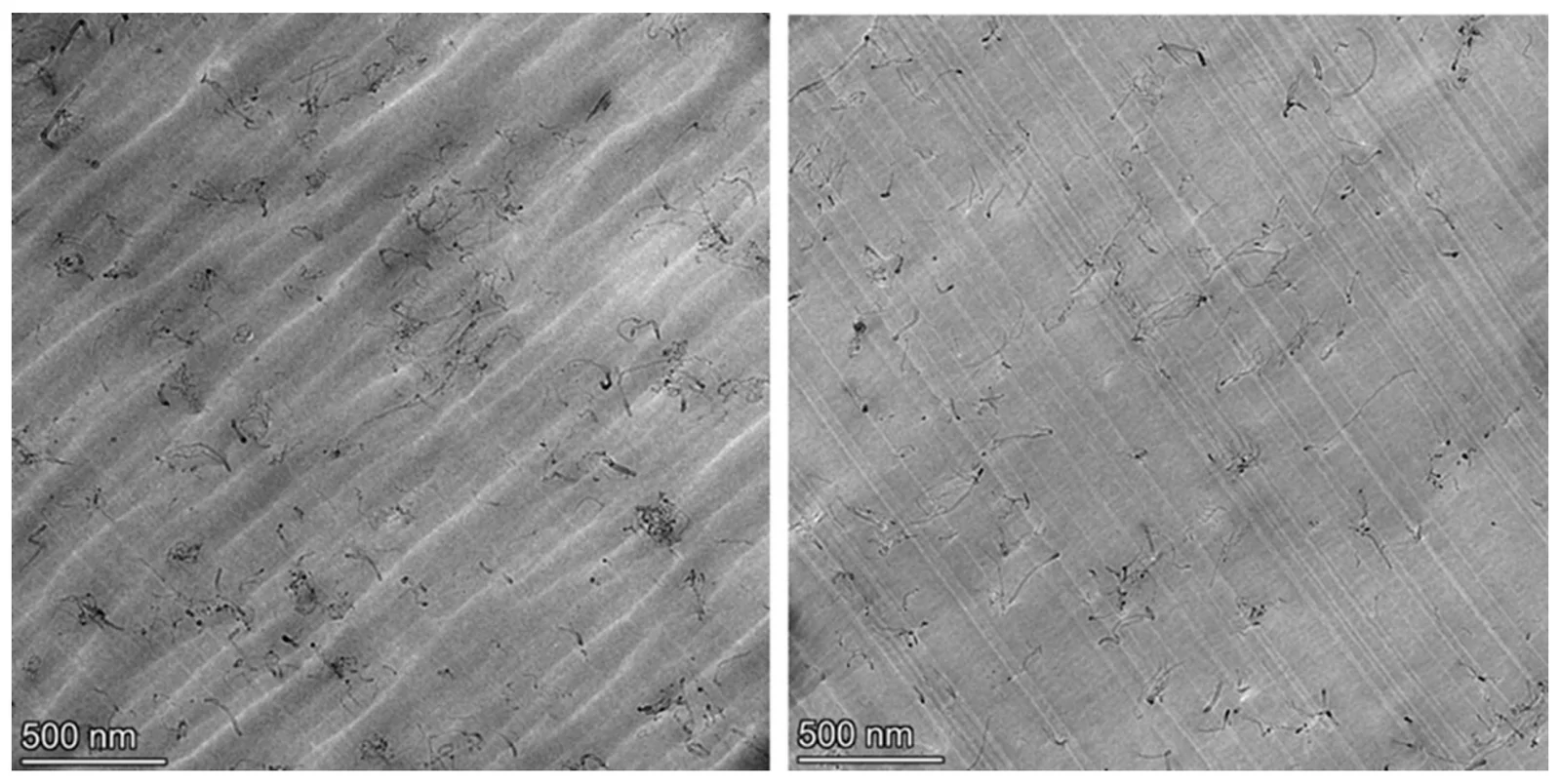
TEM images of PEEK/PEI/CNT 0.75 wt.% (**left**), and annealed PEEK/PEI/CNT 0.75 wt.% (**right**).

Figure 17 compares the electrical conductivity of PEEK/PEI/CNT nanocomposites before and after thermal annealing. In contrast to the PEEK/CNT system, both curves are nearly identical, exhibiting the same percolation threshold and overall conductivity profile. This finding indicates that annealing has little to no effect on the electrical properties of PEEK/PEI/CNT composites. TEM images of the PEEK/PEI/CNT (0.75 wt%) samples before and after annealing (Figure 18) corroborate this conclusion. In contrast to the PEEK/CNT nanocomposites, where annealing led to the formation of larger and more isolated CNT clusters, the PEEK/PEI/CNT samples exhibit minimal morphological changes. This difference stems from the distinct dispersion environments of CNTs in the two matrices. In PEEK/CNT composites, CNTs tend to form agglomerates even before annealing; thermal treatment enhances their mobility, promoting the growth of larger, more separated clusters [55,72]. In contrast, in PEEK/PEI/CNT composites, CNTs are localized within the PEI phase. Owing to the superior wettability of CNTs by PEI, the nanotubes remain uniformly dispersed, minimizing the likelihood of aggregation. As a result, the thermal energy provided during annealing is insufficient to induce significant reorganization of the CNT network or to promote additional cluster formation [72,74].

It is noteworthy that in all SAOS measurements for both annealed and unannealed PEEK/PEI/CNT samples, the storage modulus–frequency curves exhibit a plateau in the low-frequency region (0.01–0.1 rad/s), indicating the formation of a percolated nanofiller network within the polymer matrix.

### 3.5. Thermal Conductivity

**Figure 19:**
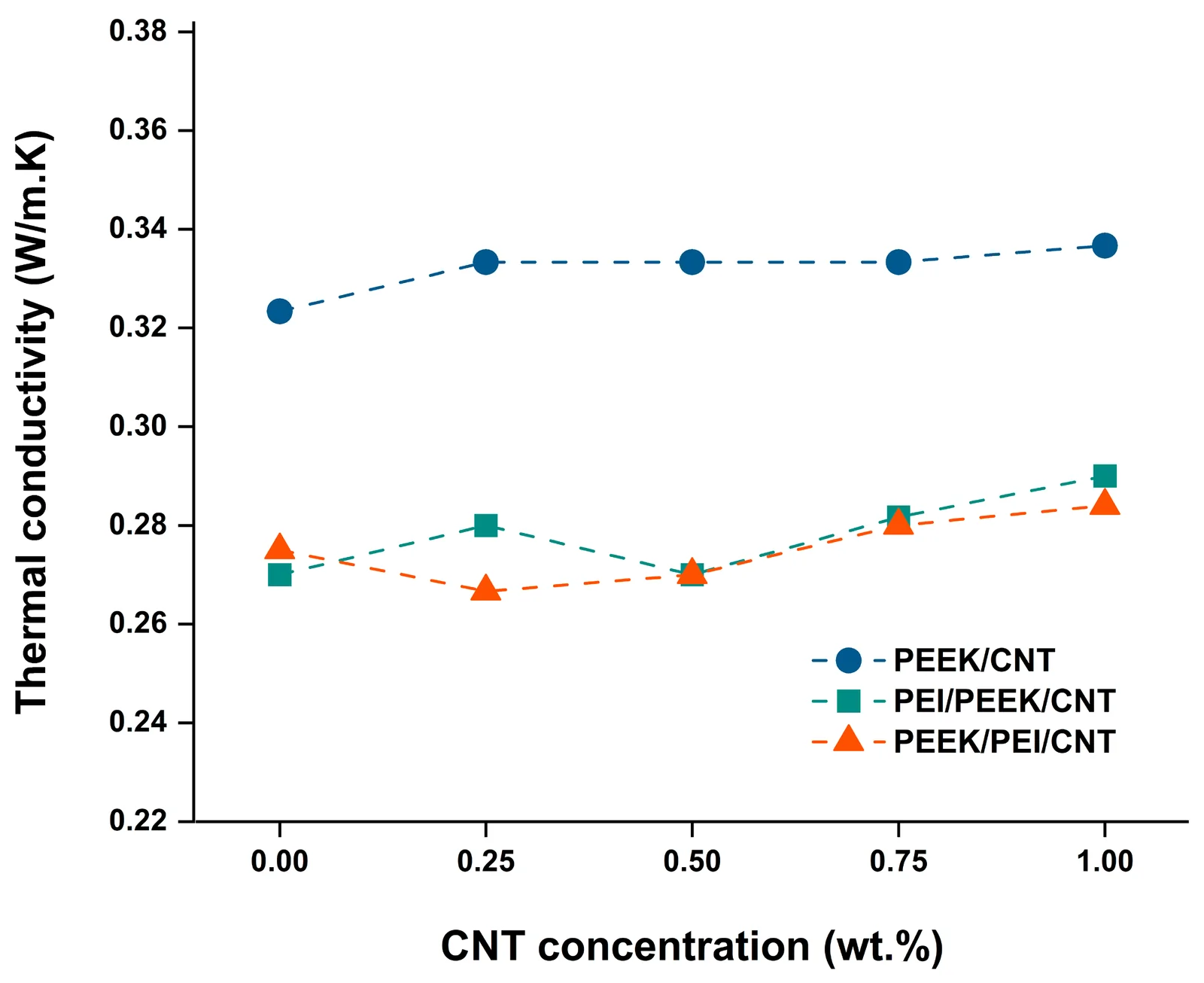
Variations in thermal conductivity of PEEK/CNT and PEEK/PEI/CNT with CNT concentration.

Figure 19 illustrates the variation in thermal conductivity of PEEK/CNT, PEEK/PEI/CNT, and PEI/PEEK/CNT composites as a function of CNT content. The blends exhibit 12–19% lower thermal conductivity compared to PEEK/CNT, depending on the CNT loading. This reduction is attributed to the inherently lower thermal conductivity of amorphous PEI (0.26 W/m·K) compared to semi-crystalline PEEK (0.32 W/m·K), as measured in the present study.

In all three systems, the thermal conductivity remains close to that of the neat matrix and increases only slightly with CNT addition, displaying nearly linear trends with very low slopes. This behavior indicates that the thermal percolation threshold has not yet been reached within the investigated CNT concentration range. The absence of a thermal percolation effect can be explained by the mechanisms governing heat transfer in polymer nanocomposites. Heat in such systems is primarily conducted through phonon transport within the polymer matrix and through a combination of phonon and free-electron transport along the CNT network. At low CNT loadings, the nanotubes are well dispersed but insufficiently interconnected to form a continuous, heat-conducting network. Consequently, the electronic contribution to heat transfer remains negligible, and the overall thermal conductivity is dominated by the intrinsic phonon-mediated conduction of the polymer matrix. The modest increase observed with increasing CNT content is thus attributed to localized phonon transfer between individual CNTs and the surrounding polymer chains [10].

The thermal conductivity values obtained in this study are also consistent with those reported for PEEK/CNT nanocomposites. Previous studies have shown that neat PEEK typically exhibits a thermal conductivity of approximately 0.25–0.30 W·m^−1^·K^−1^ and that the incorporation of low CNT contents results in only a slight increase, with values generally remaining below 0.40 W·m^−1^·K^−1^ [4,10]. Similarly, the PEEK/CNT and PEEK/PEI/CNT nanocomposites investigated here exhibited only modest increases in thermal conductivity, indicating that thermal insulation is largely preserved despite the formation of electrically conductive networks.

## 4. Conclusions

CNT localization and network formation in PEEK/CNT and PEEK/PEI/CNT nanocomposites were systematically investigated to elucidate the influence of phase morphology, processing history and thermal treatment on electrical and thermal transport behavior. Despite the strong thermodynamic preference of CNTs for the PEI phase, both systems exhibit comparable electrical percolation thresholds (0.25–0.5 wt.%), governed by distinct confinement mechanisms: crystalline exclusion in PEEK and selective CNT segregation into PEI in blends. Rheological results revealed that percolated networks form at lower CNT concentrations than those required for electrical percolation, underscoring the higher sensitivity of viscoelastic properties to the early stages of network formation. Processing parameters were found to play a decisive role in determining network continuity. Short mixing times preserved CNT interconnected agglomerates, enhancing electrical conductivity, whereas prolonged mixing promoted extensive dispersion but disrupted conductive pathways. Similarly, thermal annealing weakened the conductive network in PEEK/CNT composites through CNT agglomeration yet had minimal effect on PEEK/PEI/CNT due to the superior CNT–PEI interfacial compatibility. The sequence of masterbatch preparation was also found to be critical, as dispersing CNTs in PEI prior to blending with PEEK resulted in a more interconnected CNT network and a lower electrical percolation threshold.

Across all systems, thermal conductivity remained low and nearly unaffected by CNT addition, preserving the desirable insulating characteristics of the composites. Overall, PEEK/PEI/CNT nanocomposites provide a promising platform for multifunctional applications, achieving low percolation thresholds, tunable conductivity through processing, and stable insulation performance. These findings establish valuable structure–processing–property relationships that can guide the design of high-performance nanocomposites for aerospace technologies such as lightweight, conductive, and thermally insulating components in lunar rover systems.

## Figures and Tables

**Table 1:** Key properties of PEEK and PEI.

Polymer	Density (g/cm^3^) ^a^	T_g_ (°C) ^a^	Melting Point (°C) ^a^	Melt Viscosity (Pa.s) ^b^	Electrical Conductivity (S/cm) ^a^
PEEK	1.30	147	343	112	3.8 × 10^−17^
p-PEI	1.28	225	-	600	1 × 10^−17^

^a^: provided by supplier. ^b^: Measured by capillary rheometer at 400 °C and 1000 s^−1.^

**Table 2:** Key properties of carbon nanotube used as conductive filler.

Filler	Diameter (nm)	Length (nm)	Surface Area (m^2^/g)	Purity (%)	Synthesis Method
MWCNT (Nanocyl NC7000)	9.5	1500	250–300	90	Catalytic Chemical Vapor Deposition (CCVD)

All the data is provided by supplier (Nanocyl).

**Table 3:** Filler localization in an immiscible polymer blend according to wetting coefficient.

Wetting Coefficient	Filler Localization
ω_a_ > 1	Polymer B
−1 < ω_a_ < 1	Interphase
ω_a_ < −1	Polymer A

**Table 4:** Surface and interfacial tensions of nanocomposite components used in this study.

Material/Interface	Total Surface Tension (mN/m)	Dispersive Component (mN/m)	Polar Component (mN/m)	Interfacial Tension (mN/m)	Ref
PEEK	42.1	36.2	5.9	-	[24]
PEI	39.0	33.09	5.91	-	[38]
MWCNT	45.3	26.9	18.4	-	[39]
PEEK-PEI	-	-	-	0.14	[19]
MWCNT-PEEK	-	-	-	7.80	
MWCNT-PEI	-	-	-	7.06	

**Table 5:** Degree of crystallinity derived from DSC analysis.

Material	T_g_ Midpoint (°C)	Heat of Fusion (J/g)	Degree of Crystallinity of PEEK (%)
PEI	228	-	-
PEEK	146	43	33
PEEK/CNT 0.25%	146	43	33
PEEK/CNT 0.5%	147	41	31
Annealed PEEK/CNT 0.25%	146	39	30
Annealed PEEK/CNT 0.5%	146	41	32
PEEK-PEI	166, 204	21	40
PEEK/PEI/CNT 0.25%	167, 203	15	29
PEEK/PEI/CNT 0.50%	166, 206	14	28

**Table 6:** Degree of co-continuity measured using selective dissolution method.

Sample	Dissolved PEI (wt.%)
PEEK/PEI (40/60)	84
PEEK/PEI/CNT 0.25 wt.%	100
PEEK/PEI/CNT 1 wt.%	100
Annealed PEEK/PEI/CNT 1 wt.%	97.5
PEI/PEEK/CNT 1 wt.%	94

**Table 7:** Parameters obtained from applying linear regression on the power-law model of electrical conductivity.

Nanocomposite System	Percolation Threshold (wt.%)	t, k, R^2^
PEEK/CNT	0.25–0.5 wt.%	1.5, −5.4, 0.99
PEEK/PEI/CNT	0.25–0.5 wt.%	3.9, −7.5, 0.98
PEI/CNT	0.25–0.5 wt.%	5.8, −4.9, 0.99

## Data Availability

The original contributions presented in this study are included in the article. Further inquiries can be directed to the corresponding author.
